# An emissions-socioeconomic inventory of Chinese cities

**DOI:** 10.1038/sdata.2019.27

**Published:** 2019-02-26

**Authors:** Yuli Shan, Jianghua Liu, Zhu Liu, Shuai Shao, Dabo Guan

**Affiliations:** 1 Water Security Research Centre and School of International Development, University of East Anglia, Norwich NR4 7TJ, UK; 2 School of Urban and Regional Science, Shanghai University of Finance and Economics, Shanghai 200433, China; 3 Department of Earth System Science, Tsinghua University, Beijing 100080, China; 4 Center for Energy and Environmental Policy Research, Beijing Institute of Technology, Beijing 100081, China

**Keywords:** Climate-change policy, Climate-change mitigation, Environmental economics

## Abstract

As the centre of human activity and being under the threat of climate change, cities are considered to be major components in the implementation of climate change mitigation and CO_2_ emission reduction strategies. Inventories of cities’ emissions serve as the foundation for the analysis of emissions characteristics and policymaking. China is the world’s top energy consumer and CO_2_ emitter, and it is facing great potential harm from climate change. Consequently, China is taking increasing responsibility in the fight against global climate change. Many energy/emissions control policies have been implemented in China, most of which are designed at the national level. However, cities are at different stages of industrialization and have distinct development pathways; they need specific control policies designed based on their current emissions characteristics. This study is the first to construct emissions inventories for 182 Chinese cities. The inventories are constructed using 17 fossil fuels and 47 socioeconomic sectors. These city-level emissions inventories have a scope and format consistent with China’s national/provincial inventories. Some socioeconomic data of the cities, such as GDP, population, industrial structures, are included in the datasets as well. The dataset provides transparent, accurate, complete, comparable, and verifiable data support for further city-level emissions studies and low-carbon/sustainable development policy design. The dataset also offers insights for other countries by providing an emissions accounting method with limited data.

## Background & Summary

Cities are considered to be major components in the implementation of climate change mitigation and CO_2_ emission reduction strategies^1^. Although a mention of “city” is lacking in the Paris Agreement or the Sustainable Development Goals, as all submissions focused on the national level, climate change actions should be taken at the city level^2^.

Cities are the basic units for human activity^3^ and the main consumers of energy and emitters of CO_2_ throughout the world^4,5^. The CO_2_ emissions from energy use in cities will grow by 1.8% per year between 2006 and 2030, with the share of global CO_2_ emissions rising from 71 to 76%^6^. In China, urban energy use accounts for 85% of total emissions, which is higher than its share in the USA (80%) or Europe (69%)^7,8^. The high energy demand and high CO_2_ emissions of cities not only increase climate change concerns and environmental pressure but also increase residents’ health problems through air pollution^9^. Both coastal and interior cities are facing danger from extreme weather, geological hazards, urban waterlogging, etc. Thus, cities are motivated to fight against climate change.

Although climate policies are usually designed at the national level, they are implemented at the city level. Without support from local city governments, national policies cannot be effectively executed. Considering that cities have different natural resource endowments and development pathways, each should have specific emission reduction actions that are designed based on that city’s unique emission characteristics. In China, this is particularly true. There are over 330 cities in China, and they are at different stages of industrialization, with distinct development pathways. Therefore, cities are the key components in climate change policymaking, and many low-carbon projects and actions have been taken at the city level, such as the Local Governments for Sustainability (ICLEI) and the C40 Cities Climate Leadership Group (C40).

Understanding the emissions characteristics of cities is the foundation of any further city-level climate change actions. Compared to studies focused on national and provincial emissions accounts, far fewer have focused on city-level emissions, and those that do have methods limitations and geographical restrictions.

First, previous studies on city-level emissions have severe methodological weaknesses and limitations. Most previous city-level greenhouse gas emissions inventories were calculated using a bottom-up approach, i.e., by using energy consumption data from surveys of several sectors^10–12^. The sectors were set differently between studies, making the cities’ CO_2_ emissions inconsistent and not comparable across studies, as well as inconsistent with the national and regional emission inventories. In addition, some studies used spatial and geographical analysis^13,14^, night-time light imagery^15,16^, or economic models^17,18^ to account for city emissions. These models can only estimate the overall CO_2_ emissions of a city. They cannot exactly determine the contributions of emission sources (i.e., energy types or socioeconomic sectors).

Second, most of the previous studies on city-level emissions focused on megacities from developed countries with consistent and transparent data sources, especially US cities^19–23^. Currently, city-level emissions are being studied from a more international perspective by analysing more global cities, especially cities from developing countries^24–30^. Restricted by data availability, the CO_2_ emissions from Chinese cities are far behind in their documentation. Sugar, *et al*.^31^ reported emissions for Beijing, Tianjin, and Shanghai in 2006 and compared the three cities’ emissions with those of ten other global cities. Wang, *et al*.^10^ discussed the CO_2_ emissions from 12 Chinese megacities, most of which are provincial capital cities. Dhakal^8^ examined the energy consumption and CO_2_ emissions of all Chinese provincial cities. Zhou, *et al*.^32^ and Xu, *et al*.^33^ account for the CO_2_ emissions of specific city clusters, such as the Guangdong Bay cities and cities in the central plain. Ramaswami, *et al*.^34^ in the cited study and a follow-up study developed a comprehensive emission database including the scope 1 and scope 2 CO_2_ emissions of 233 prefecture-level and 637 county-level cities in China^35^.

Thus, the previous assessments of city-level emissions either focused on total emissions (or combined emissions for several sectors) or on megacities with consistent and systematic energy statistics. Previous analyses of the bottom-up sector-based emissions of cities are inconsistent with national and regional emission inventories, making multi-scale emission studies unavailable. Additionally, such general emission data cannot support detailed city-level emission analysis and related emission reduction policy making.

The dataset in this study provides detailed emissions inventories for 182 Chinese cities. The inventories are constructed for 17 types of fossil fuel and 47 socioeconomic sectors that are consistent with the System of National Accounts. Additional socioeconomic indexes for the cities are included in the dataset. The dataset has been re-used in our latest study^1^ and will facilitate further city-level emissions studies and low-carbon/sustainable development policy design.

## Methods

### City boundaries and emission scopes

This dataset provides the emissions and socioeconomic inventories of 182 Chinese cities; these cities cover 82% (33,880 billion yuan) of the country’s GDP (41,303 billion yuan), 64% (860 million) of the population (1,341 million), and 35% (3.4 million km^2^) of the land area (9.6 million km^2^) in 2010^36^. Most of the studied cities are located east of the Heihe-Tengchong line, where 96% of China’s population lives on 43% of the land. The 182 cities are selected based on data availability.

The term ‘city’ here refers to administrative prefecture-level city rather than to a built-up city. Accordingly, the CO_2_ emissions calculated in this dataset are Intergovernmental Panel on Climate Change (IPCC) administrative territorial CO_2_ emissions, referring to emissions “taking place within national (including administered) territories and offshore areas over which the country has jurisdiction (page overview.5)”^37^. We exclude the emissions induced by international aviation and shipping^38^. Unlike production- or consumption-based emissions^17^, the administrative territorial scope quantifies the direct emissions induced by human activities within a regional boundary. That is, territorial emissions provide the data baseline for emission-related studies and regional carbon control.

The emission inventories include two components: CO_2_ emitted from fossil fuel combustion (energy-related emissions) and CO_2_ emitted from industrial production (process-related emissions). Process-related emissions refers to CO_2_ emitted from industrial raw materials during chemical reactions, such as CO_2_ escaping during calcium carbonate (*CaCO*_3_) calcination in cement production.

The cities’ emissions inventories are uniform with China’s national and provincial emission inventories in scope, format, and data sources^39^, making them comparable.

### Emissions calculation and inventory construction

The energy-related emissions are calculated based on 17 fuels (shown in Table 1) and 47 socioeconomic sectors (shown in Table 2). The 17 types of fossil fuels are selected based on China’s official energy statistical system^36^. There are 29 energy types used in the system: 26 are fossil fuels, one is electricity, one is heat, and one is other energy. As our study only accounts for the direct emissions from fossil fuel burning within one city boundary (the IPCC administrative territorial scope), the inventories exclude the indirect emissions induced by electricity and heat use. The CO_2_ emissions related to electricity and heat generation, therefore, are calculated based on fuel inputs and allocated to the power plants. We also assume that there is no, or little, CO_2_ emitted from other energy uses. Some of the fossil fuels share similar carbon content and have very low consumption volumes; we merge them in the emission accounts^39^. The 47 socioeconomic sectors are set according to the System of National Accounts^40^.

Energy-related CO_2_ emissions are calculated based on the mass balance theory;^41^ see Equation 1.

(1)CEij=ADij×NCVi×CCi×Oij

where *CE*_*ij*_ represents the CO_2_ emissions induced by the combustion of fuel *i* in sector *j*, *AD*_*ij*_ (activity data) represents fossil fuel combustion by fuel and sector. The emission factor (*ton CO*_2_/*ton*) is composed of a specific heat value factor- *NCV*_*i*_ (*J*/*ton*) multiplied by the carbon content per unit heat value-*CC*_*i*_ (*ton CO*_2_/*J*) and oxygenation efficiency-*O*_*ij*_ (quantified as percentage). Specifically, *NCV*_*i*_ refers to the heat value produced per physical unit of fossil fuel *i* combusted, *CC*_*i*_ is the carbon content emitted per unit heat value when combusting per physical unit of fossil fuel *i*, while *O*_*ij*_ stands for the oxidation ratio of the fossil fuel combusted.

The emission factors (*NCV*_*i*_, *CC*_*i*_, and *O*_*ij*_) have been published by international institutions, including the IPCC and the United Nations (UN; governmental agencies in China such as the National Bureau of Statistics of China (NBS) and the National Development and Reform Commission of China (NDRC);^42^ and previous studies such as the Multi-resolution Emission Inventory for China (MEIC)^43^, Liu, *et al*.^44^. Liu, *et al*.^44^ re-evaluated the carbon content of raw coal samples from 4,243 state-owned Chinese coal mines and found that the emission factors for Chinese coal are, on average, 40% lower than the default values recommended by the IPCC. After comparing Liu, *et al*.^44^ emissions factors with eight different sources, our previous study finds that Liu, *et al*.^44^ emission factors are relatively lower than others (shown in Table 3 (available online only)). The seven sets of emission factors are collected from IPCC, NBS, NDRC, NC1994, NC2005, MEIC, UN-China, and UN-average. Generally, coal-related fuels have a larger range than oil- and gas-related fuels. Liu, *et al*.^44^’s re-evaluated emission factors have already been widely used by many studies and institutions to calculate China’s emission inventory, including China’s third official emission inventory 2012^45^ . Thus, this study uses the above-mentioned updated emission factors. Table 1 gives the net caloric value (*NCV*_*i*_) and carbon content (*CC*_*i*_). Table 4 (available online only) shows the sector-specific oxygenation efficiency (*O*_*ij*_), which considers sector discrepancies in technical level^39^.

The process-related CO_2_ emissions (*CE*_*t*_) are calculated in Equation 2^41^. We include seven industrial processes, including cement production (for approximately 70% of the total process-related emissions in China^45,46^), lime production (the 2^nd^ largest emissions source^47^), ammonia production, soda ash production, ferrochromium production, silicon metal production, and unclassified ferro-production. The process-related emissions are allocated to the corresponding sectors in the emission inventory. Cement and lime-related emissions are allocated to the sector “Non-metal Mineral Products”; ammonia and soda ash-related emissions are allocated to the sector “Raw Chemical Materials and Chemical Products”; Ferrochromium, silicon metal, and unclassified ferro-related emissions are allocated to the sector “Smelting and Pressing of Ferrous Metals”.

(2)CEt=ADt×EFt

*AD*_*t*_ and *EF*_*t*_ in the equation refer to industrial production (activity data) and emission factors, respectively. The emission factors of industrial processes are collected from IPCC^41^ and NDRC^42^, as shown in Table 5.

The cities’ CO_2_ emissions matrices (namely, inventories) are created as 19 columns and 48 rows. Seventeen fossil fuel-related emissions, process-related emissions and total emissions are represented by 19 columns, while 47 rows correspond to the 47 socioeconomic sectors. Each element of the matrices is identified as the CO_2_ emissions from fossil fuel combustion/industrial production in the corresponding sector. An inventory of Beijing is given in Table 6 (available online only) as an example.

These methods on emission inventory construction are expanded version of descriptions in our related work^39^. MATLAB R2014a is used to construct the cities’ emission inventories. We provided the MATLAB code in the Supplementary Information. We also provided the activity data of the cities for additional data transparency and verifiability (see “China city-level Energy inventory, 2010”, Data Citation 1). Researchers will be able to use the MATLAB code and energy inventories to recalculate the emission inventories for the cities or replicate to other cities.

### Activity data collection

Fossil fuel combustion, i.e., the activity data for energy-related emission accounts, includes two parts: the energy inputs for electricity/heat generation and the total final consumption. Other inputs for energy transformation, such as coal cleaning or petroleum refineries, transfer the carbon element from one fuel to another. These processes emit little CO_2_. Following our previous emissions inventories constructed for China and its provinces^39^, fossil fuel combustion can be collected from a region’s energy balance table (EBT) and final energy consumption can be captured by the industrial sector (*Energy*_*ij*_). The EBT provides each fossil fuel’s transformation and final consumption in farming, industry, construction, three service sectors, and households (rural and urban). As the entire industry sector consists of 40 sub-sectors, *Energy*_*ij*_ presents the sectoral consumption of fossil fuel for the industry sector.

Generally, the EBT and *Energy*_*ij*_ can be found in a city’s statistical yearbook. However, due to the poor data quality of city-level statistics, not all cities’ yearbooks publish the EBT or *Energy*_*ij*_. We developed a series of methods in our previous study to estimate missing data^48^:

1. EBT: Very few cities have EBT in their statistical yearbooks. We scale down the corresponding provincial EBT to obtain the city table. We use each sector’s GDP to estimate farming, construction, and three service sectors, assuming that the city has the same farming/construction/service energy intensity as its province. We also use the urban/rural population to estimate the urban/rural household energy estimation on the premise that the city has the same per capita residential energy consumption as its province. The GDP and population data are collected from statistical yearbooks for the cities and their corresponding provinces.
2. *Energy*_*ij*_: Some cities only provide *Energy*_*ij*_ from enterprises of above-designated-size (ADS). ADS enterprises are defined as enterprises with prime operating revenue over 20 or 5 million yuan for different cities. ADS enterprises account for 50 to 90% (roughly) of one city’s total industrial output. We use the ADS industrial output ratio (calculated as the whole-industry output divided by the ADS enterprises’ output) to scale up ADS *Energy*_*ij*_ and obtain sectoral fossil fuel consumption at the whole-industry scale.

As for cement production, the cities’ statistical yearbooks provide total cement production or production from ADS enterprises. We then scaled up the ADS cement production by the ADS industrial output ratio to obtain the total cement production.

The raw activity data are collected through a “crowd-sourcing” working mode implemented in the Applied Energy Summer School 2017 and 2018. Over 100 students joined the summer school and participated in data collection. The summer school will be held annually in the future, and more researchers will contribute to and update city-level data collection.

These methods on city-level data estimation and collection are expanded version of descriptions in our related work^48^.

### Socioeconomic indexes

This study collects several socioeconomic indexes for the 182 cities from the “China City Statistical Yearbook”^49^, including:

1. population, in 10 thousand;
2. employed population, in 10 thousand;
3. employed population in sectors (primary industry; mining; manufacturing, electric power, gas and water production and supply; construction; transport, storage and post; information transmission, computer services and software industry; wholesale and retail trade; hotel and catering services; financial intermediation; real estate; leasing and business services; scientific research, technical services and geological exploration; water, environmental and public facilities management; resident services and other services; education; health, social security and social welfare; culture, sports and entertainment; public administration and social organization), in 10 thousand;
4. area, in square kilometres;
5. built up area, in square kilometres;
6. gross domestic product (GDP), in 10 thousand yuan;
7. primary industry, secondary industry, and tertiary industry’s share in GDP, in %;
8. industrial output, in 10 thousand yuan.

The socioeconomic indexes (as shown in Table 7 (available online only) and “China city-level socioeconomic inventory, 2010”, Data Citation 1) can be used to explore the drivers and characteristics of cities’ emissions.

## Data Records

A total of 365 data records (emissions-socioeconomic inventories) are contained in the datasets. Of these,

- 182 are emissions inventories for cities (2010) [“China city-level emissions inventory, 2010”, Data Citation 1];
- 182 are energy inventories for cities (2010) [“China city-level energy inventory, 2010”, Data Citation 1];
- 1 is a socioeconomic inventory for cities (2010) [“China city-level socioeconomic inventory, 2010”, Data Citation 1];

The cities’ CO_2_ emissions inventories are constructed at an IPCC territorial administrative scope, including both energy-related emissions (from fossil fuel combustion) and process-related emissions (from cement production). The socioeconomic inventory presents GDP, population, employed population (with structure), GDP (with structure), and area of the 182 cities.

## Technical Validation

### Uncertainties

CO_2_ emissions inventories gather the contributions of economic activity to total CO_2_ emissions for a given time period and area. Inventories are critical to many environmental decision-making processes and scientific goals. Policymaking and scientific research require reliable inventories to ensure the effectiveness of the policy process. In both types of applications, it is important to understand the uncertainty in emissions inventories. Additionally, uncertainty analysis can improve the accuracy of emissions accounts. Regarding the city-level CO_2_ emissions inventories in this article, the literature shows that uncertainty regarding the process-related emissions in cement production is low. The inventories’ uncertainty mainly depends on energy-related emissions part^44,50^. The contributing sources of uncertainty for energy-related emissions accounting are associated with emission factors, activity data and other estimation parameters *(Volume 1, Chapter 3, Page 6)*”^41^. The uncertainty induced by emissions factors and energy activity data are both quantified for the cities’ emission inventories.

#### Uncertainties in activity data and emission factors

China’s energy data are of relatively poor quality compared with those of developed countries, especially city-level data. The literature also shows that the uncertainties range widely from sector to sector. The coefficient of variation (CV; the standard deviation divided by the mean) is used to quantify the uncertainty. According to a field survey led by previous studies, the fossil fuel consumed in China’s power generation sector has the lowest CV (5%)^51,52^, compared with primary industry (30%)^53^, other manufacturing sectors (10%), construction (10%)^41,54^, transportation sector (16%)^55^, and residential energy use (20%)^41^. The sources of uncertainties could lie in the opaqueness in China’s statistical systems, especially on the “*statistical approach on data collection, reporting and validation (Page 673)*”^56^ and the dependence of China’s statistics departments on other government departments. Such uncertainties result in a large gap between China’s national fossil fuel consumption data and the aggregated provincial data. To cover the gap, China has adjusted its energy data three times since 2004, resulting in a gap between the latest national fossil fuel consumption data and provincial aggregated data of 5%^57^. The gap between city-level aggregated energy consumption and the national overall data could be even larger.

Previous studies have debated China’s emission factors^58–61^. The range of emission factors across different sources is as high as 40%. This study collects emission factors from Liu, *et al*.^44^, which measured them based on a broad investigation of China’s fuel quality. Based on the statistical analysis of surveyed fuel quality, the CVs of coal-, oil-, and gas-related fuels are estimated as 3, 1, and 2%, respectively.

#### Monte Carlo simulations

Monte Carlo methods are used to simulate the uncertainties resulting from both fossil fuel combustion and emissions factors to estimate the overall uncertainty of the emissions^41^. Monte Carlo simulations select random values for the emission factor and activity data (fossil fuel consumption) from within their individual normal probability (density) functions and calculate the corresponding emission values (chapter 6 IPCC^41^). To perform Monte Carlo simulations, we first set up probability density functions for each input variable (emission factor and activity data). Both variables are assumed to follow a normal distribution^44^. Then, we randomly sample both the activity data and the emission factors 20,000 times and obtain 20,000 CO_2_ emission estimations. The uncertainties are obtained at a 97.5% confidence level and are calculated as the 97.5% confidence intervals of the estimates.

This article finds that the average uncertainties in the cities’ total CO_2_ emissions range from −;3.65 to 3.67% at a 97.5% confidence level (±47.5% confidence interval around the estimate). Hegang in Heilongjiang has the highest uncertainties in emissions of (−5.83, 5.86%), while Huizhou in Guangxi has the lowest value of (−0.91, 0.91%).

### Limitations and future work

The cities’ emission inventories have some limitations that could lead to more uncertainty. Although these uncertainties may not be large enough to quantify, they are an indispensable component of the emission inventories’ uncertainties. First, this study only takes the energy-related and process-related emissions from seven industrial production processes into account in the emission accounts, and emissions emitted by other sources is missing, such as “agriculture”, “land-use change and forestry”, “waste”, and other industrial processes. Thus, the analysis incomplete. In the future, we will expand the emission scope to achieve more complete inventories for cities. Second, the cities’ emission factors for fossil fuels and industrial processes are substituted by national average emission factors during the process of accounting for cities’ CO_2_ emissions, resulting in inaccuracy. We hope that specific city-level emissions factors could be updated in the future to increase the accuracy of our results. If not, in our future research, we could employ provincial emission factors to obtain a more accurate emission inventory for the provinces. Third, due to the poor data quality for the cities, the EBTs of most cities are a downscaled version of the provincial table, assuming that the cities have the same sectoral energy intensity and per capita residential energy consumption with their provinces. Such assumptions bring additional uncertainties to cities’ emission inventories. In the future, a consistent time-series emission inventory dataset for Chinese cities will be completed. We will integrate the bottom-up estimations (calculated based on survey data from enterprises)^14^ and satellite observations to achieve more emission accounts for these cities. More specifically, the high-resolution bottom-up emissions and satellite images can confirm some of the cities’ emission sources (i.e. some super-emitting points). The night-light data will also be used to verify our top-down emissions inventories^16,62^.

## Additional information

**How to cite this article**: Rashid, H. *et al*. An emissions-socioeconomic inventory of Chinese cities. *Sci. Data*. 6:190027 https://doi.org/10.1038/sdata.2019.27 (2019).

**Publisher’s note**: Springer Nature remains neutral with regard to jurisdictional claims in published maps and institutional affiliations.

## Supplementary Material



Supplementary Information

## Figures and Tables

**Table 1:** Fossil fuels in the city-level emissions inventories and emissions factors.

No. (i) Unit	Fuels in China’s Energy Statistics	Fuels in this study	*NCV*_*i*_	*CC*_*i*_
			PJ/10^4^ *tonnes,* 10^8^ *m*^3^	tonne C/*TJ*
1	Raw coal	Raw coal	0.21	26.32
2	Cleaned coal	Cleaned coal	0.26	26.32
3	Other washed coal	Other washed coal	0.15	26.32
4	Briquettes	Briquette	0.18	26.32
		Gangue		
5	Coke	Coke	0.28	31.38
6	Coke oven gas	Coke over gas	1.61	21.49
7	Blast furnace gas	Other gas	0.83	21.49
	Converter gas			
	Other gas			
8	Other coking products	Other coking products	0.28	27.45
9	Crude Oil	Crude oil	0.43	20.08
10	Gasoline	Gasoline	0.44	18.9
11	Kerosene	Kerosene	0.44	19.6
12	Diesel oil	Diesel oil	0.43	20.2
13	Fuel oil	Fuel oil	0.43	21.1
14	Naphtha	Other petroleum products	0.51	17.2
	Lubricants			
	Paraffin			
	White spirit			
	Bitumen asphalt			
	Petroleum coke			
	Other petroleum products			
15	Liquefied petroleum gas (LPG)	LPG	0.47	20
16	Refinery gas	Refinery gas	0.43	20.2
17	Nature gas	Nature gas	3.89	15.32

**Table 2:** Sectors’ definition of the emission inventories.

No. (j)	Socioeconomic sectors	Category	
1	Farming, Forestry, Animal Husbandry, Fishery and Water Conservancy	The primary industry	
2	Coal Mining and Dressing	Energy production	Manufacturing industries
3	Petroleum and Natural Gas Extraction	Energy production	
4	Ferrous Metals Mining and Dressing	Energy production	
5	Nonferrous Metals Mining and Dressing	Energy production	
6	Non-metal Minerals Mining and Dressing	Energy production	
7	Other Minerals Mining and Dressing	Energy production	
8	Logging and Transport of Wood and Bamboo	Light manufacturing	
9	Food Processing	Light manufacturing	
10	Food Production	Light manufacturing	
11	Beverage Production	Light manufacturing	
12	Tobacco Processing	Light manufacturing	
13	Textile Industry	Light manufacturing	
14	Garments and Other Fibre Products	Light manufacturing	
15	Leather, Furs, Down and Related Products	Light manufacturing	
16	Timber Processing, Bamboo, Cane, Palm Fibre & Straw Products	Light manufacturing	
17	Furniture Manufacturing	Light manufacturing	
18	Papermaking and Paper Products	Light manufacturing	
19	Printing and Record Medium Reproduction	Light manufacturing	
20	Cultural, Educational and Sports Articles	Light manufacturing	
21	Petroleum Processing and Coking	Energy production	
22	Raw Chemical Materials and Chemical Products	Heavy manufacturing	
23	Medical and Pharmaceutical Products	Light manufacturing	
24	Chemical Fibre	Heavy manufacturing	
25	Rubber Products	Heavy manufacturing	
26	Plastic Products	Heavy manufacturing	
27	Non-metal Mineral Products	Heavy manufacturing	
28	Smelting and Pressing of Ferrous Metals	Heavy manufacturing	
29	Smelting and Pressing of Nonferrous Metals	Heavy manufacturing	
30	Metal Products	Heavy manufacturing	
31	Ordinary Machinery	Heavy manufacturing	
32	Equipment for Special Purposes	Heavy manufacturing	
33	Transportation Equipment manufacturing	Heavy manufacturing	
34	Electric Equipment and Machinery	High-tech industry	
35	Electronic and Telecommunications Equipment	High-tech industry	
36	Instruments, Meters, Cultural and Office Machinery	High-tech industry	
37	Other Manufacturing Industry	High-tech industry	
38	Scrap and waste	High-tech industry	
39	Production and Supply of Electric Power, Steam and Hot Water	Energy production	
40	Production and Supply of Gas	Energy production	
41	Production and Supply of Tap Water	Heavy manufacturing	
42	Construction	Construction	
43	Transportation, Storage, Post and Telecommunication Services	Services sectors	
44	Wholesale, Retail Trade and Catering Services		
45	Other Service Sectors		
46	Urban Resident Energy Usage	Household	
47	Rural Resident Energy Usage		

**Table 3:** Fuel’s emission factors from other sources.

		IPCC	NBS	NDRC	NC1994	NC2005	MEIC	UN-China	UN average	Liu *et al.*’s nature
Net caloric value (PJ/10 thousand tonnes, 100 million cu.m.)	Raw Coal	0.28	0.21	0.21	0.21	0.22	0.19	0.21	0.29	0.21
	Cleaned Coal	0.27	0.26	0.23	0.24	0.23	0.26	0.21	0.29	0.26
	Other Washed Coal	0.27	0.15	0.23	0.21	0.23	0.15	0.21	0.29	0.15
	Briquettes	0.26	0.18	0.17	0.20	0.17	0.18	0.21	0.29	0.18
	Coke	0.28	0.28	0.28	0.28	0.28	0.28	0.26	0.26	0.28
	Coke Oven Gas	1.88	1.63	1.74	1.63	1.74	1.67	1.88	1.88	1.61
	Other Gas	1.88	0.84	1.58	0.84	1.58	0.52	1.88	1.88	0.83
	Other Coking Products	0.43	0.28	0.28	0.28	0.28	0.42	0.43	0.43	0.28
	Crude Oil	0.42	0.42	0.43	0.42	0.43	0.42	0.42	0.42	0.43
	Gasoline	0.44	0.43	0.45	0.45	0.45	0.43	0.45	0.45	0.44
	Kerosene	0.44	0.43	0.45	0.45	0.45	0.43	0.43	0.43	0.44
	Diesel Oil	0.43	0.43	0.43	0.45	0.43	0.43	0.42	0.42	0.43
	Fuel Oil	0.40	0.42	0.40	0.40	0.40	0.42	0.40	0.40	0.43
	LPG	0.47	0.50	0.47	0.47	0.47	0.50	0.46	0.46	0.51
	Refinery Gas	0.50	0.46	0.46	0.40	0.46	0.46	0.42	0.42	0.47
	Other Petroleum Products	0.40	0.42	0.45	0.40	0.45	0.42	0.42	0.42	0.43
	Natural Gas	3.44	3.89	3.89	3.90	3.89	3.89	3.44	3.44	3.89
Carbon content (C/TJ)	Raw Coal	25.80	26.37	26.37	24.26	25.83	25.80	25.80	25.80	26.32
	Cleaned Coal	26.80	25.41	25.41	26.35	27.82	25.80	26.80	26.80	26.32
	Other Washed Coal	26.80	25.41	25.41	24.26	27.82	25.80	26.80	26.80	26.32
	Briquettes	25.80	33.56	33.56	24.26	33.56	25.80	25.80	25.80	26.32
	Coke	29.20	29.42	29.42	29.50	28.84	25.52	29.20	29.20	31.38
	Coke Oven Gas	12.10	13.58	13.58	20.00	14.00	15.16	12.10	12.10	21.49
	Other Gas	12.10	12.20	12.20	12.10	12.20	15.16	12.10	12.10	21.49
	Other Coking Products	25.80	29.50	29.50	25.80	20.00	19.91	25.80	25.80	27.45
	Crude Oil	20.00	20.08	20.08	20.00	20.08	19.91	20.00	20.00	20.08
	Gasoline	18.90	18.90	18.90	18.90	18.90	19.91	18.90	18.90	18.90
	Kerosene	19.50	19.60	19.60	19.60	19.60	19.91	19.50	19.50	19.60
	Diesel Oil	20.20	20.20	20.20	20.20	20.20	19.91	20.20	20.20	20.20
	Fuel Oil	21.10	21.10	21.10	21.10	21.10	19.91	21.10	21.10	21.10
	LPG	17.20	17.20	17.20	17.20	17.20	19.91	17.20	17.20	17.20
	Refinery Gas	15.70	18.20	18.20	15.70	18.20	19.91	15.70	15.70	20.00
	Other Petroleum Products	20.00	20.00	20.00	20.00	20.00	19.91	20.00	20.00	20.20
	Natural Gas	15.30	15.32	15.32	15.30	15.32	15.16	15.30	15.30	15.32
Oxygenation efficiency	Raw Coal	0.98	0.94	0.94	0.90	0.92	1.00	1.00	1.00	0.92
	Cleaned Coal	0.98	0.98	0.98	0.90	0.92	1.00	1.00	1.00	0.92
	Other Washed Coal	0.98	0.98	0.98	0.90	0.92	1.00	1.00	1.00	0.92
	Briquettes	0.98	0.90	0.90	0.90	0.90	1.00	1.00	1.00	0.92
	Coke	0.98	0.93	0.93	0.97	0.93	1.00	1.00	1.00	0.92
	Coke Oven Gas	0.99	0.99	0.99	0.99	0.99	1.00	1.00	1.00	0.92
	Other Gas	0.99	0.99	0.99	0.99	0.99	1.00	1.00	1.00	0.92
	Other Coking Products	0.99	0.93	0.93	0.97	0.93	1.00	1.00	1.00	0.92
	Crude Oil	0.99	0.98	0.98	0.98	0.98	1.00	1.00	1.00	0.98
	Gasoline	0.99	0.98	0.98	0.98	0.98	1.00	1.00	1.00	0.98
	Kerosene	0.99	0.98	0.98	0.98	0.98	1.00	1.00	1.00	0.98
	Diesel Oil	0.99	0.98	0.98	0.98	0.98	1.00	1.00	1.00	0.98
	Fuel Oil	0.99	0.98	0.98	0.98	0.98	1.00	1.00	1.00	0.98
	LPG	0.99	0.99	0.99	0.99	0.99	1.00	1.00	1.00	0.98
	Refinery Gas	0.99	0.99	0.99	0.99	0.99	1.00	1.00	1.00	0.98
	Other Petroleum Products	0.99	0.98	0.98	0.98	0.98	1.00	1.00	1.00	0.98
	Natural Gas	0.99	0.99	0.99	0.99	0.99	1.00	1.00	1.00	0.99
Emission factor (ton CO2/ton)	Raw Coal	2.61	1.90	1.90	1.67	1.94	1.80	1.98	2.77	1.83
	Cleaned Coal	2.57	2.41	2.12	2.13	2.17	2.49	2.05	2.88	2.31
	Other Washed Coal	2.57	1.41	2.12	1.66	2.17	1.46	2.05	2.88	1.33
	Briquettes	2.39	1.97	1.93	1.60	1.93	1.68	1.98	2.77	1.60
	Coke	2.96	2.85	2.85	2.99	2.79	2.66	2.82	2.82	2.96
	Coke Oven Gas	8.26	8.04	8.55	11.84	8.85	9.30	8.34	8.34	11.67
	Other Gas	8.26	3.73	6.98	3.70	6.98	2.91	8.34	8.34	6.02
	Other Coking Products	4.03	2.86	2.86	2.61	1.94	3.05	4.07	4.07	2.59
	Crude Oil	3.07	3.02	3.08	3.01	3.08	3.05	3.10	3.10	3.10
	Gasoline	3.05	2.93	3.04	3.04	3.04	3.14	3.11	3.11	2.99
	Kerosene	3.10	3.04	3.15	3.15	3.15	3.14	3.09	3.09	3.10
	Diesel Oil	3.15	3.10	3.15	3.25	3.15	3.11	3.15	3.15	3.12
	Fuel Oil	3.09	3.17	3.05	3.05	3.05	3.05	3.13	3.13	3.26
	LPG	2.95	3.13	2.95	2.95	2.95	3.66	2.87	2.87	3.15
	Refinery Gas	2.82	3.04	3.04	2.29	3.04	3.36	2.41	2.41	3.38
	Other Petroleum Products	2.90	3.01	3.24	2.89	3.24	3.05	3.12	3.12	3.12
	Natural Gas	1.91	2.17	2.17	2.17	2.17	2.16	1.93	1.93	2.16

**Table 4:** Oxygenation efficiency of fossil fuels combusted in sectors.

Sectors	Raw Coal	Cleaned Coal	Other Washed Coal	Briquettes	Coke	Coke Oven Gas	Other Gas	Other Coking Products	Crude Oil	Gasoline	Kerosene	Diesel Oil	Fuel Oil	LPG	Refinery Gas	Other Petroleum Products	Natural Gas
Farming, Forestry, Animal Husbandry, Fishery and Water Conservancy	83%	83%	83%	83%	89%	91%	91%	89%	96%	96%	96%	96%	96%	97%	97%	96%	98%
Coal Mining and Dressing	82%	82%	82%	82%	89%	91%	91%	89%	96%	96%	96%	96%	96%	97%	97%	96%	98%
Petroleum and Natural Gas Extraction	82%	82%	82%	82%	89%	91%	91%	89%	96%	96%	96%	96%	96%	97%	97%	96%	98%
Ferrous Metals Mining and Dressing	82%	82%	82%	82%	89%	91%	91%	89%	96%	96%	96%	96%	96%	97%	97%	96%	98%
Nonferrous Metals Mining and Dressing	82%	82%	82%	82%	89%	91%	91%	89%	96%	96%	96%	96%	96%	97%	97%	96%	98%
Non-metal Minerals Mining and Dressing	82%	82%	82%	82%	89%	91%	91%	89%	96%	96%	96%	96%	96%	97%	97%	96%	98%
Other Minerals Mining and Dressing	82%	82%	82%	82%	89%	91%	91%	89%	96%	96%	96%	96%	96%	97%	97%	96%	98%
Logging and Transport of Wood and Bamboo	82%	82%	82%	82%	89%	91%	91%	89%	96%	96%	96%	96%	96%	97%	97%	96%	98%
Food Processing	80%	80%	80%	80%	89%	91%	91%	89%	96%	96%	96%	96%	96%	97%	97%	96%	98%
Food Production	80%	80%	80%	80%	89%	91%	91%	89%	96%	96%	96%	96%	96%	97%	97%	96%	98%
Beverage Production	80%	80%	80%	80%	89%	91%	91%	89%	96%	96%	96%	96%	96%	97%	97%	96%	98%
Tobacco Processing	80%	80%	80%	80%	89%	91%	91%	89%	96%	96%	96%	96%	96%	97%	97%	96%	98%
Textile Industry	82%	82%	82%	82%	89%	91%	91%	89%	96%	96%	96%	96%	96%	97%	97%	96%	98%
Garments and Other Fibre Products	82%	82%	82%	82%	89%	91%	91%	89%	96%	96%	96%	96%	96%	97%	97%	96%	98%
Leather, Furs, Down and Related Products	82%	82%	82%	82%	89%	91%	91%	89%	96%	96%	96%	96%	96%	97%	97%	96%	98%
Timber Processing, Bamboo, Cane, Palm Fibre & Straw Products	80%	80%	80%	80%	89%	91%	91%	89%	96%	96%	96%	96%	96%	97%	97%	96%	98%
Furniture Manufacturing	80%	80%	80%	80%	89%	91%	91%	89%	96%	96%	96%	96%	96%	97%	97%	96%	98%
Papermaking and Paper Products	80%	80%	80%	80%	89%	91%	91%	89%	96%	96%	96%	96%	96%	97%	97%	96%	98%
Printing and Record Medium Reproduction	80%	80%	80%	80%	89%	91%	91%	89%	96%	96%	96%	96%	96%	97%	97%	96%	98%
Cultural, Educational and Sports Articles	80%	80%	80%	80%	89%	91%	91%	89%	96%	96%	96%	96%	96%	97%	97%	96%	98%
Petroleum Processing and Coking	83%	83%	83%	83%	89%	91%	91%	89%	96%	96%	96%	96%	96%	97%	97%	96%	98%
Raw Chemical Materials and Chemical Products	85%	85%	85%	85%	89%	91%	91%	89%	96%	96%	96%	96%	96%	97%	97%	96%	98%
Medical and Pharmaceutical Products	85%	85%	85%	85%	89%	91%	91%	89%	96%	96%	96%	96%	96%	97%	97%	96%	98%
Chemical Fibre	85%	85%	85%	85%	89%	91%	91%	89%	96%	96%	96%	96%	96%	97%	97%	96%	98%
Rubber Products	85%	85%	85%	85%	89%	91%	91%	89%	96%	96%	96%	96%	96%	97%	97%	96%	98%
Plastic Products	85%	85%	85%	85%	89%	91%	91%	89%	96%	96%	96%	96%	96%	97%	97%	96%	98%
Non-metal Mineral Products	90%	90%	90%	90%	89%	91%	91%	89%	96%	96%	96%	96%	96%	97%	97%	96%	98%
Smelting and Pressing of Ferrous Metals	84%	84%	84%	84%	89%	91%	91%	89%	96%	96%	96%	96%	96%	97%	97%	96%	98%
Smelting and Pressing of Nonferrous Metals	84%	84%	84%	84%	89%	91%	91%	89%	96%	96%	96%	96%	96%	97%	97%	96%	98%
Metal Products	82%	82%	82%	82%	89%	91%	91%	89%	96%	96%	96%	96%	96%	97%	97%	96%	98%
Ordinary Machinery	82%	82%	82%	82%	89%	91%	91%	89%	96%	96%	96%	96%	96%	97%	97%	96%	98%
Equipment for Special Purposes	82%	82%	82%	82%	89%	91%	91%	89%	96%	96%	96%	96%	96%	97%	97%	96%	98%
Transportation Equipment	82%	82%	82%	82%	89%	91%	91%	89%	96%	96%	96%	96%	96%	97%	97%	96%	98%
Electric Equipment and Machinery	82%	82%	82%	82%	89%	91%	91%	89%	96%	96%	96%	96%	96%	97%	97%	96%	98%
Electronic and Telecommunications Equipment	82%	82%	82%	82%	89%	91%	91%	89%	96%	96%	96%	96%	96%	97%	97%	96%	98%
Instruments, Meters, Cultural and Office Machinery	82%	82%	82%	82%	89%	91%	91%	89%	96%	96%	96%	96%	96%	97%	97%	96%	98%
Other Manufacturing Industry	82%	82%	82%	82%	89%	91%	91%	89%	96%	96%	96%	96%	96%	97%	97%	96%	98%
Scrap and Waste	87%	87%	87%	87%	89%	91%	91%	89%	96%	96%	96%	96%	96%	97%	97%	96%	98%
Production and Supply of Electric Power, Steam and Hot Water	87%	87%	87%	87%	89%	91%	91%	89%	96%	96%	96%	96%	96%	97%	97%	96%	98%
Production and Supply of Gas	82%	82%	82%	82%	89%	91%	91%	89%	96%	96%	96%	96%	96%	97%	97%	96%	98%
Production and Supply of Tap Water	82%	82%	82%	82%	89%	91%	91%	89%	96%	96%	96%	96%	96%	97%	97%	96%	98%
Construction	83%	83%	83%	83%	89%	91%	91%	89%	96%	96%	96%	96%	96%	97%	97%	96%	98%
Transportation, Storage, Post and Telecommunication Services	74%	74%	74%	74%	89%	91%	91%	89%	96%	96%	96%	96%	96%	97%	97%	96%	98%
Wholesale, Retail Trade and Catering Services	74%	74%	74%	74%	89%	91%	91%	89%	96%	96%	96%	96%	96%	97%	97%	96%	98%
Others	74%	74%	74%	74%	89%	91%	91%	89%	96%	96%	96%	96%	96%	97%	97%	96%	98%
Urban Household Energy Use	74%	74%	74%	74%	89%	91%	91%	89%	96%	96%	96%	96%	96%	97%	97%	96%	98%
Rural Household Energy Use	74%	74%	74%	74%	89%	91%	91%	89%	96%	96%	96%	96%	96%	97%	97%	96%	98%

**Table 5:** 7 industrial processes and emissions factors.

*No.*	*Industry process*	*Emission factors*	*Allocation sectors*
1	Ammonia production	1.5000	Raw Chemical Materials and Chemical Products
2	Soda Ash production	0.4150	Raw Chemical Materials and Chemical Products
3	Cement production	0.4985	Non-metal Mineral Products
4	Lime production	0.6830	Non-metal Mineral Products
5	Ferrochromium production	1.3000	Smelting and Pressing of Ferrous Metals
6	Silicon metal production	4.3000	Smelting and Pressing of Ferrous Metals
7	Ferro-unclassified production	4.0000	Smelting and Pressing of Ferrous Metals

**Table 6:** Emissions inventory of Beijing in 2010.

Unit: million tonnes	Raw Coal	Cleaned Coal	Other Washed Coal	Briquettes	Coke	Coke Oven Gas	Other Gas	Other Coking Products	Crude Oil	Gasoline	Kerosene	Diesel Oil	Fuel Oil	LPG	Refinery Gas	Other Petroleum Products	Natural Gas	Non-fossil Heat	Non-fossil Electricity	Other Energy	Process	Total
Total Consumption	39.6	0.0	0.1	0.5	6.3	0.9	0.0	0.0	0.0	10.9	11.9	7.3	0.5	1.3	2.5	0.4	15.2	0.0	0.0	0.0	5.2	102.6
Farming, Forestry, Animal Husbandry, Fishery and Water Conservancy	0.8	0.0	0.0	0.0	0.0	0.0	0.0	0.0	0.0	0.1	0.0	0.2	0.0	0.0	0.0	0.0	0.0	0.0	0.0	0.0	0.0	1.1
Coal Mining and Dressing	0.0	0.0	0.0	0.0	0.0	0.0	0.0	0.0	0.0	0.0	0.0	0.0	0.0	0.0	0.0	0.0	0.0	0.0	0.0	0.0	0.0	0.0
Petroleum and Natural Gas Extraction	0.0	0.0	0.0	0.0	0.0	0.0	0.0	0.0	0.0	0.0	0.0	0.0	0.0	0.0	0.0	0.0	0.0	0.0	0.0	0.0	0.0	0.0
Ferrous Metals Mining and Dressing	1.2	0.0	0.0	0.0	1.0	0.1	0.0	0.0	0.0	0.1	0.0	0.2	0.0	0.0	0.3	0.0	0.3	0.0	0.0	0.0	0.0	3.3
Nonferrous Metals Mining and Dressing	0.0	0.0	0.0	0.0	0.0	0.0	0.0	0.0	0.0	0.0	0.0	0.0	0.0	0.0	0.0	0.0	0.0	0.0	0.0	0.0	0.0	0.0
Nonmetal Minerals Mining and Dressing	0.0	0.0	0.0	0.0	0.0	0.0	0.0	0.0	0.0	0.0	0.0	0.0	0.0	0.0	0.0	0.0	0.0	0.0	0.0	0.0	0.0	0.0
Other Minerals Mining and Dressing	0.0	0.0	0.0	0.0	0.0	0.0	0.0	0.0	0.0	0.0	0.0	0.0	0.0	0.0	0.0	0.0	0.0	0.0	0.0	0.0	0.0	0.0
Logging and Transport of Wood and Bamboo	0.0	0.0	0.0	0.0	0.0	0.0	0.0	0.0	0.0	0.0	0.0	0.0	0.0	0.0	0.0	0.0	0.0	0.0	0.0	0.0	0.0	0.0
Food Processing	0.1	0.0	0.0	0.0	0.1	0.0	0.0	0.0	0.0	0.0	0.0	0.0	0.0	0.0	0.0	0.0	0.0	0.0	0.0	0.0	0.0	0.2
Food Production	0.0	0.0	0.0	0.0	0.0	0.0	0.0	0.0	0.0	0.0	0.0	0.0	0.0	0.0	0.0	0.0	0.0	0.0	0.0	0.0	0.0	0.1
Beverage Production	0.1	0.0	0.0	0.0	0.1	0.0	0.0	0.0	0.0	0.0	0.0	0.0	0.0	0.0	0.0	0.0	0.0	0.0	0.0	0.0	0.0	0.4
Tobacco Processing	0.0	0.0	0.0	0.0	0.0	0.0	0.0	0.0	0.0	0.0	0.0	0.0	0.0	0.0	0.0	0.0	0.0	0.0	0.0	0.0	0.0	0.0
Textile Industry	0.0	0.0	0.0	0.0	0.0	0.0	0.0	0.0	0.0	0.0	0.0	0.0	0.0	0.0	0.0	0.0	0.0	0.0	0.0	0.0	0.0	0.1
Garments and Other Fiber Products	0.0	0.0	0.0	0.0	0.0	0.0	0.0	0.0	0.0	0.0	0.0	0.0	0.0	0.0	0.0	0.0	0.0	0.0	0.0	0.0	0.0	0.1
Leather, Furs, Down and Related Products	0.0	0.0	0.0	0.0	0.0	0.0	0.0	0.0	0.0	0.0	0.0	0.0	0.0	0.0	0.0	0.0	0.0	0.0	0.0	0.0	0.0	0.0
Timber Processing, Bamboo, Cane, Palm Fiber & Straw Products	0.0	0.0	0.0	0.0	0.0	0.0	0.0	0.0	0.0	0.0	0.0	0.0	0.0	0.0	0.0	0.0	0.0	0.0	0.0	0.0	0.0	0.0
Furniture Manufacturing	0.0	0.0	0.0	0.0	0.0	0.0	0.0	0.0	0.0	0.0	0.0	0.0	0.0	0.0	0.0	0.0	0.0	0.0	0.0	0.0	0.0	0.0
Papermaking and Paper Products	0.0	0.0	0.0	0.0	0.0	0.0	0.0	0.0	0.0	0.0	0.0	0.0	0.0	0.0	0.0	0.0	0.0	0.0	0.0	0.0	0.0	0.1
Printing and Record Medium Reproduction	0.0	0.0	0.0	0.0	0.0	0.0	0.0	0.0	0.0	0.0	0.0	0.0	0.0	0.0	0.0	0.0	0.0	0.0	0.0	0.0	0.0	0.0
Cultural, Educational and Sports Articles	0.0	0.0	0.0	0.0	0.0	0.0	0.0	0.0	0.0	0.0	0.0	0.0	0.0	0.0	0.0	0.0	0.0	0.0	0.0	0.0	0.0	0.0
Petroleum Processing and Coking	0.0	0.0	0.0	0.0	0.0	0.0	0.0	0.0	0.0	0.0	0.0	0.0	0.0	0.0	0.0	0.0	0.0	0.0	0.0	0.0	0.0	0.0
Raw Chemical Materials and Chemical Products	0.4	0.0	0.0	0.0	0.3	0.0	0.0	0.0	0.0	0.0	0.0	0.0	0.0	0.0	0.1	0.0	0.1	0.0	0.0	0.0	0.0	0.9
Medical and Pharmaceutical Products	0.0	0.0	0.0	0.0	0.0	0.0	0.0	0.0	0.0	0.0	0.0	0.0	0.0	0.0	0.0	0.0	0.0	0.0	0.0	0.0	0.0	0.1
Chemical Fiber	0.0	0.0	0.0	0.0	0.0	0.0	0.0	0.0	0.0	0.0	0.0	0.0	0.0	0.0	0.0	0.0	0.0	0.0	0.0	0.0	0.0	0.0
Rubber Products	0.0	0.0	0.0	0.0	0.0	0.0	0.0	0.0	0.0	0.0	0.0	0.0	0.0	0.0	0.0	0.0	0.0	0.0	0.0	0.0	0.0	0.0
Plastic Products	0.0	0.0	0.0	0.0	0.0	0.0	0.0	0.0	0.0	0.0	0.0	0.0	0.0	0.0	0.0	0.0	0.0	0.0	0.0	0.0	0.0	0.1
Nonmetal Mineral Products	0.8	0.0	0.0	0.0	0.6	0.1	0.0	0.0	0.0	0.0	0.0	0.1	0.0	0.0	0.2	0.0	0.2	0.0	0.0	0.0	5.2	7.2
Smelting and Pressing of Ferrous Metals	0.0	0.0	0.0	0.0	0.0	0.0	0.0	0.0	0.0	0.0	0.0	0.0	0.0	0.0	0.0	0.0	0.0	0.0	0.0	0.0	0.0	0.0
Smelting and Pressing of Nonferrous Metals	0.0	0.0	0.0	0.0	0.0	0.0	0.0	0.0	0.0	0.0	0.0	0.0	0.0	0.0	0.0	0.0	0.0	0.0	0.0	0.0	0.0	0.0
Metal Products	0.0	0.0	0.0	0.0	0.0	0.0	0.0	0.0	0.0	0.0	0.0	0.0	0.0	0.0	0.0	0.0	0.0	0.0	0.0	0.0	0.0	0.1
Ordinary Machinery	0.0	0.0	0.0	0.0	0.0	0.0	0.0	0.0	0.0	0.0	0.0	0.0	0.0	0.0	0.0	0.0	0.0	0.0	0.0	0.0	0.0	0.1
Equipment for Special Purposes	0.0	0.0	0.0	0.0	0.0	0.0	0.0	0.0	0.0	0.0	0.0	0.0	0.0	0.0	0.0	0.0	0.0	0.0	0.0	0.0	0.0	0.1
Transportation Equipment	0.1	0.0	0.0	0.0	0.1	0.0	0.0	0.0	0.0	0.0	0.0	0.0	0.0	0.0	0.0	0.0	0.0	0.0	0.0	0.0	0.0	0.3
Electric Equipment and Machinery	0.0	0.0	0.0	0.0	0.0	0.0	0.0	0.0	0.0	0.0	0.0	0.0	0.0	0.0	0.0	0.0	0.0	0.0	0.0	0.0	0.0	0.1
Electronic and Telecommunications Equipment	0.0	0.0	0.0	0.0	0.0	0.0	0.0	0.0	0.0	0.0	0.0	0.0	0.0	0.0	0.0	0.0	0.0	0.0	0.0	0.0	0.0	0.0
Instruments, Meters, Cultural and Office Machinery	0.0	0.0	0.0	0.0	0.0	0.0	0.0	0.0	0.0	0.0	0.0	0.0	0.0	0.0	0.0	0.0	0.0	0.0	0.0	0.0	0.0	0.0
Other Manufacturing Industry	0.0	0.0	0.0	0.0	0.0	0.0	0.0	0.0	0.0	0.0	0.0	0.0	0.0	0.0	0.0	0.0	0.0	0.0	0.0	0.0	0.0	0.0
Scrap and waste	0.0	0.0	0.0	0.0	0.0	0.0	0.0	0.0	0.0	0.0	0.0	0.0	0.0	0.0	0.0	0.0	0.0	0.0	0.0	0.0	0.0	0.0
Production and Supply of Electric Power, Steam and Hot Water	27.4	0.0	0.1	0.4	3.9	0.6	0.0	0.0	0.0	0.3	0.0	0.8	0.4	0.0	1.6	0.4	6.8	0.0	0.0	0.0	0.0	42.7
Production and Supply of Gas	0.0	0.0	0.0	0.0	0.0	0.0	0.0	0.0	0.0	0.0	0.0	0.0	0.0	0.0	0.0	0.0	0.0	0.0	0.0	0.0	0.0	0.0
Production and Supply of Tap Water	0.0	0.0	0.0	0.0	0.0	0.0	0.0	0.0	0.0	0.0	0.0	0.0	0.0	0.0	0.0	0.0	0.0	0.0	0.0	0.0	0.0	0.0
Construction	0.3	0.0	0.0	0.0	0.0	0.0	0.0	0.0	0.0	0.3	0.0	1.2	0.0	0.0	0.0	0.0	0.1	0.0	0.0	0.0	0.0	1.9
Transportation, Storage, Post and Telecommunication Services	0.3	0.0	0.0	0.0	0.0	0.0	0.0	0.0	0.0	1.2	11.9	3.9	0.0	0.0	0.0	0.0	0.5	0.0	0.0	0.0	0.0	17.9
Wholesale, Retail Trade and Catering Services	0.6	0.0	0.0	0.0	0.0	0.0	0.0	0.0	0.0	0.6	0.0	0.3	0.0	0.4	0.0	0.0	1.0	0.0	0.0	0.0	0.0	2.9
Others	3.0	0.0	0.0	0.1	0.0	0.0	0.0	0.0	0.0	1.4	0.0	0.5	0.0	0.1	0.0	0.0	3.9	0.0	0.0	0.0	0.0	8.9
Urban	1.1	0.0	0.0	0.0	0.0	0.0	0.0	0.0	0.0	6.5	0.0	0.0	0.0	0.5	0.0	0.0	2.1	0.0	0.0	0.0	0.0	10.2
Rural	3.0	0.0	0.0	0.0	0.0	0.0	0.0	0.0	0.0	0.2	0.0	0.0	0.0	0.2	0.0	0.0	0.1	0.0	0.0	0.0	0.0	3.5

**Table 7:** Emissions-socioeconomic indexes of the cities

City-Ename	City-Cname	CO2 emissions	Population	Employed population	Employed population in “primary industry”	Employed population in "mining"	Employed population in "manufacturing"	Employed population in "electric power, gas and water production and supply"	Employed population in "construction"	Employed population in "transport, storage and post"	Employed population in "information transmission, computer services and software industry"	Employed population in "wholesale and retail trade"	Employed population in "hotel and catering services"	Employed population in "financial intermediation"	Employed population in "real estate"	Employed population in "leasing and business services"	Employed population in "scientific research, technical services and geological exploration"	Employed population in "water, environmental and public facilities management"	Employed population in "resident services and other services"	Employed population in "education"	Employed population in "health, social security and social welfare"	Employed population in "culture, sports and entertainment"	Employed population in "public administration and social organization"	Area	Built up area	GDP	Primay industry's share in GDP	Secondary industry's share in GDP	Tertiary industry's share in GDP	Industrial output
		million tonnes	10 thousand	10 thousand	10 thousand	10 thousand	10 thousand	10 thousand	10 thousand	10 thousand	10 thousand	10 thousand	10 thousand	10 thousand	10 thousand	10 thousand	10 thousand	10 thousand	10 thousand	10 thousand	10 thousand	10 thousand	10 thousand	Square kilometers	Square kilometers	10 thousand yuan	%	%	%	10 thousand yuan
Beijing	北京	102.6	1257.80	646.63	3.23	4.54	100.55	6.80	39.35	51.00	41.73	55.38	27.96	27.24	31.54	77.81	45.74	8.76	7.44	40.56	20.67	15.25	41.08	16411	1186	141135800	0.88	24.01	75.11	136998388
Tianjin	天津	132.0	984.85	205.65	0.71	8.96	75.30	3.27	10.21	12.50	2.24	12.39	4.83	6.95	3.61	6.95	6.47	3.55	6.86	16.43	8.98	1.75	13.69	11760	687	92244600	1.58	52.47	45.95	167518155
Shijiazhuang	河北石家庄	119.5	989.16	84.15	0.41	0.67	23.28	2.66	4.50	5.67	0.91	6.09	1.28	4.44	0.41	0.85	2.23	1.69	0.32	12.34	3.91	1.42	11.07	15848	203	34010186	10.87	48.63	40.51	56553423
Tangshan	河北唐山	194.0	735.00	84.02	3.03	10.42	24.81	2.76	5.14	3.87	0.65	4.12	0.62	3.77	0.56	0.84	0.40	1.66	0.12	9.07	3.43	0.48	8.27	13472	234	44691588	9.44	58.14	32.42	75450331
Handan	河北邯郸	129.6	963.50	56.57	0.25	6.59	9.22	2.71	3.79	2.42	0.53	2.31	0.36	2.48	0.31	0.47	0.95	1.49	0.04	10.75	2.87	0.52	8.51	12062	111	23615569	13.04	54.21	32.75	41073243
Zhangjiakou	河北张家口	53.2	465.97	33.72	0.55	2.32	6.54	1.33	1.74	1.08	0.46	1.47	0.28	1.35	0.63	0.28	0.41	0.75	0.08	5.80	1.95	0.23	6.47	36873	84	9664158	15.83	42.96	41.21	8934836
Taiyuan	山西太原	87.0	365.50	84.64	0.33	8.40	24.69	1.57	8.09	7.71	1.10	3.51	1.91	2.56	0.51	1.44	3.16	1.60	0.39	7.54	2.94	1.47	5.72	6963	245	17780539	1.70	44.91	53.39	19946527
Yangquan	山西阳泉	33.0	130.78	23.74	0.04	11.29	2.16	0.84	0.96	0.83	0.10	0.88	0.11	0.67	0.16	0.39	0.16	0.23	0.02	1.88	0.69	0.14	2.19	4570	52	4293774	1.53	59.46	39.01	5426118
Changzhi	山西长治	57.4	331.54	37.30	0.31	9.39	7.95	1.04	1.32	1.06	0.29	1.38	0.20	1.48	0.10	0.54	0.30	0.67	0.01	4.64	1.44	0.39	4.79	13896	59	9202336	4.37	65.38	30.24	14332828
Jincheng	山西晋城	34.6	216.23	27.15	0.15	11.79	2.31	0.72	0.62	0.67	0.24	1.61	0.21	1.00	0.07	0.26	0.15	0.43	0.04	2.71	1.05	0.20	2.92	9425	41	7305428	4.20	63.60	32.19	8511244
Shuozhou	山西朔州	27.2	159.07	17.89	0.87	3.88	1.36	0.80	1.38	0.31	0.23	1.21	0.17	0.56	0.12	0.55	0.10	0.20	0.08	1.95	0.51	0.11	3.50	11066	36	6701476	6.05	56.56	37.39	8285136
Jinzhong	山西晋中	60.7	320.96	35.06	0.18	7.33	6.75	1.12	2.11	0.85	0.34	1.50	0.42	1.73	0.19	0.43	0.56	0.54	0.02	4.52	1.62	0.32	4.53	16392	39	7638366	8.50	54.76	36.74	10302374
Xinzhou	山西忻州	24.2	307.55	22.70	0.33	2.64	1.81	0.44	1.25	0.77	0.39	1.50	0.29	0.89	0.07	0.31	0.22	0.45	0.04	4.33	1.47	0.20	5.30	25117	30	4374561	11.25	44.59	44.15	3930339
Hohhot	内蒙古呼和浩特	69.0	229.56	31.50	0.36	0.02	5.84	1.47	1.50	1.44	0.84	1.13	0.70	1.99	0.21	0.93	1.37	1.55	0.30	4.68	1.68	0.93	4.56	17224	166	18657116	4.90	36.39	58.71	11885198
Baotou	内蒙古包头	119.2	219.80	32.59	0.31	0.95	12.62	1.28	2.10	1.37	0.61	1.22	0.69	1.89	0.25	0.28	0.65	0.87	0.11	3.12	1.27	0.32	2.68	27768	183	24608100	2.70	54.11	43.19	24122422
Wuhai	内蒙古乌海	21.6	53.00	9.52	0.05	2.80	1.30	0.48	1.58	0.23	0.13	0.13	0.02	0.32	0.10	0.06	0.09	0.28	0.01	0.70	0.30	0.07	0.87	1754	63	3911235	0.95	71.72	27.33	5453200
Chifeng	内蒙古赤峰	38.1	457.74	30.50	1.95	3.49	3.64	1.30	1.77	0.89	0.34	0.76	0.19	1.16	0.14	0.41	0.40	0.52	0.07	6.55	1.98	0.31	4.63	90021	81	10862293	16.33	51.24	32.43	12658407
Tongliao	内蒙古通辽	61.2	318.70	24.34	5.82	1.35	2.15	0.79	1.60	0.60	0.35	0.57	0.16	0.74	0.25	0.12	0.32	0.73	0.03	4.27	1.29	0.24	2.96	59535	66	11766183	15.15	58.62	26.23	18097485
Ordos	内蒙古鄂尔多斯	131.6	152.38	17.31	0.47	3.05	2.93	0.82	0.07	0.32	0.29	0.25	0.05	0.79	0.03	0.13	0.19	0.85	0.01	2.53	0.79	0.24	3.50	86752	113	26432300	2.68	58.69	38.63	26810700
Ulanqab	内蒙古乌兰察布	43.1	287.02	14.62	0.36	0.18	1.06	0.90	0.66	0.71	0.30	0.63	0.12	0.75	0.10	0.12	0.29	0.56	0.02	2.82	0.85	0.24	3.95	54492	35	5676016	16.55	52.28	31.17	6774228
Shenyang	辽宁沈阳	63.4	719.60	110.42	0.99	2.17	30.40	3.22	5.48	10.70	1.52	5.66	2.06	4.28	2.27	4.61	4.59	3.15	1.37	11.45	6.04	1.68	8.78	12980	412	50175427	4.64	50.42	44.94	96125255
Dalian	辽宁大连	73.9	586.44	94.20	0.97	0.22	40.88	1.71	4.76	5.30	2.42	3.61	2.33	5.03	2.88	1.93	1.59	1.41	0.36	7.82	4.11	0.82	6.05	12574	390	51581621	6.69	50.88	42.43	77018355
Benxi	辽宁本溪	67.0	154.60	23.21	0.19	1.73	8.36	0.83	2.01	1.00	0.24	0.65	0.06	0.91	0.50	0.16	0.23	0.65	0.04	2.10	1.33	0.19	2.03	8411	107	8603675	5.04	62.30	32.66	15113534
Dandong	辽宁丹东	10.1	241.36	21.22	0.30	0.51	4.90	0.90	1.70	1.14	0.42	0.98	0.32	0.67	0.65	0.21	0.72	0.80	0.06	2.51	1.28	0.25	2.90	15290	53	7288908	13.73	51.20	35.07	8650865
Fuxin	辽宁阜新	34.8	192.38	17.54	0.44	4.58	1.67	0.81	0.89	0.42	0.25	0.58	0.08	0.75	0.15	0.22	0.26	0.40	0.04	2.45	1.10	0.24	2.21	10355	76	3788656	24.46	41.82	33.72	4477708
Changchun	吉林长春	61.2	758.89	92.81	1.33	1.24	28.33	2.64	5.24	3.40	2.13	4.49	1.88	3.38	2.15	3.10	4.04	2.60	0.41	12.59	4.68	1.65	7.53	20604	394	33290329	7.59	51.66	40.74	58841613
Jilin	吉林吉林	41.3	434.03	33.25	1.64	1.06	10.28	1.44	1.48	0.99	0.27	0.82	0.17	1.40	0.19	0.13	0.47	0.86	0.05	5.12	2.18	0.37	4.33	27126	166	18006376	10.80	49.76	39.44	21041551
Siping	吉林四平	21.8	340.55	21.14	1.03	0.27	5.85	0.68	0.58	0.57	0.32	0.35	0.06	0.88	0.27	0.06	0.54	0.85	0.03	3.84	1.87	0.32	2.77	14080	51	7795527	27.13	42.75	30.12	10364734
Liaoyuan	吉林辽源	18.4	123.75	8.83	0.36	2.44	0.81	0.40	0.13	0.21	0.09	0.12	0.02	0.41	0.08	0.07	0.13	0.19	0.02	1.35	0.70	0.11	1.19	5140	46	4101426	10.43	56.18	33.39	5856489
Baicheng	吉林白城	7.6	202.64	18.51	3.40	0.01	1.55	0.42	0.53	0.54	0.23	0.74	0.17	0.58	0.14	0.17	0.34	1.46	0.39	3.16	1.36	0.25	3.07	25745	38	4451802	18.74	45.26	36.00	2592456
Yanbian	吉林延边	14.3																												
Harbin	黑龙江哈尔滨	68.9	992.02	135.17	5.50	0.71	33.70	3.79	11.47	10.83	2.29	10.21	2.45	4.76	2.73	2.70	4.29	2.89	1.95	16.09	6.36	1.76	10.69	53068	359	36648538	11.26	37.78	50.96	23047364
Qiqihaer	黑龙江齐齐哈尔	45.6	568.11	41.97	7.65	0.02	9.46	1.47	1.80	4.54	0.50	0.96	0.05	1.50	0.45	0.24	0.60	1.33	0.10	4.85	2.25	0.37	3.83	42469	135	8804569	21.81	40.63	37.55	8320561
Jixi	黑龙江鸡西	44.1	189.20	27.66	6.70	7.74	1.89	0.86	1.03	0.89	0.20	1.04	0.25	0.67	0.07	0.09	0.26	0.49	0.24	1.93	0.78	0.16	2.37	22531	79	4194931	25.55	42.31	32.14	2889386
Hegang	黑龙江鹤岗	43.2	109.10	25.89	8.55	8.01	1.39	0.47	0.44	0.63	0.14	0.96	0.33	0.42	0.05	0.01	0.08	0.55	0.31	1.35	0.63	0.14	1.43	14659	43	2509870	26.45	46.60	26.95	2844194
Shuangyashan	黑龙江双鸭山	18.8	151.58	32.81	14.61	5.07	1.67	0.83	0.64	1.10	0.23	1.12	0.38	0.48	0.11	0.12	0.11	0.60	0.69	1.76	0.81	0.16	2.32	23209	59	3963504	30.34	44.78	24.88	3618786
Daqing	黑龙江大庆	106.8	279.80	51.89	0.34	11.85	7.18	2.33	5.98	1.57	0.56	1.70	0.21	1.35	0.47	0.05	5.06	0.34	2.99	4.09	1.94	0.35	3.53	21219	207	29000642	3.28	82.24	14.48	32879601
Yichunhlj	黑龙江伊春	8.4	126.95	18.21	10.23	0.26	2.28	0.64	0.62	0.33	0.26	0.14	0.04	0.40	0.09	0.04	0.15	0.21	0.01	0.65	0.42	0.10	1.34	32759	161	2024407	30.34	39.25	30.41	1945740
Jiamusi	黑龙江佳木斯	11.5	253.78	27.63	9.56	0.06	1.94	0.81	1.79	1.30	0.20	1.34	0.33	0.77	0.10	0.19	0.30	0.91	0.52	2.95	1.38	0.18	3.00	32704	94	5124563	28.58	26.13	45.30	3139380
Heihe	黑龙江黑河	6.6	174.21	29.46	16.30	0.33	0.86	0.69	0.66	1.14	0.08	1.00	0.44	0.66	0.05	0.28	0.20	0.61	0.47	1.92	0.89	0.15	2.73	82164	19	2610994	44.78	17.12	38.09	946708
Shanghai	上海	187.5	1412.32	392.87	1.54	0.09	141.32	5.41	11.34	36.30	6.71	26.43	11.67	23.63	11.15	18.64	23.25	5.86	3.29	26.14	16.72	4.71	18.67	6340	866	171659800	0.66	42.05	57.28	301144067
Nanjing	江苏南京	141.1	632.42	125.64	0.41	0.32	47.41	1.78	10.08	8.87	2.43	8.71	3.90	3.12	2.03	3.97	4.36	1.77	0.33	11.54	4.72	1.73	8.16	6587	619	51306500	2.77	45.37	51.85	86094998
Wuxi	江苏无锡	73.8	466.56	82.95	0.24		45.43	1.44	5.29	2.36	0.82	3.36	1.64	2.64	0.63	1.65	1.20	0.92	0.17	6.36	3.30	0.57	4.93	4627	231	57933000	1.81	55.39	42.80	129710811
Xuzhou	江苏徐州	78.6	972.89	61.82	1.86	8.10	12.25	1.77	1.73	6.09	0.60	2.52	0.30	2.31	0.35	0.28	0.81	1.31	0.05	10.34	3.99	0.42	6.74	11259	239	29421394	9.61	50.67	39.71	51129660
Changzhou	江苏常州	48.2	360.80	38.16	0.12	0.04	16.88	0.72	1.19	1.76	0.40	1.32	0.59	1.62	0.42	0.61	0.75	0.82	0.03	4.72	2.42	0.32	3.43	4372	153	30448900	3.28	55.30	41.43	73960851
Suzhoujs	江苏苏州	173.0	637.66	130.87	0.15		90.06	1.43	3.20	2.29	1.09	2.93	1.77	3.69	0.90	0.91	0.73	1.27	0.10	7.67	4.69	0.70	7.29	8488	329	92289100	1.69	56.93	41.38	246516665
Nantong	江苏南通	42.0	762.92	63.11	1.13		30.26	1.00	5.13	1.88	0.61	1.54	0.35	2.89	0.47	0.80	0.43	0.75	0.05	7.12	3.46	0.36	4.88	8001	125	34656700	7.68	55.07	37.25	73831630
Lianyungang	江苏连云港	20.0	497.73	34.00	1.87	0.98	8.78	0.92	2.99	2.04	0.40	1.19	0.23	1.47	0.46	0.24	0.51	0.75	0.03	5.18	1.92	0.20	3.84	7500	120	11933100	15.30	45.68	39.02	19362814
Huaian	江苏淮安	23.7	538.74	39.28	1.13	0.37	14.67	0.79	2.99	1.04	0.26	1.11	0.19	1.41	0.33	0.63	0.21	1.23	0.01	5.94	1.94	0.27	4.76	10072	120	13880700	14.12	46.62	39.26	24391100
Yancheng	江苏盐城	24.9	816.12	51.91	2.42	0.61	16.37	0.87	6.52	1.23	0.53	1.79	0.41	2.54	0.36	1.25	0.41	0.89	0.07	7.38	2.65	0.37	5.24	16972	89	23327600	16.04	47.01	36.95	39383400
Yangzhou	江苏扬州	31.4	459.12	40.09	0.09	1.80	13.78	0.41	6.99	0.91	0.52	0.95	0.44	1.24	0.31	0.45	0.47	0.66	0.07	4.91	2.04	0.21	3.84	6591	82	22294884	7.24	55.14	37.62	57533421
Zhenjiang	江苏镇江	44.2	270.71	37.26	0.15	0.18	18.35	0.79	1.88	1.44	0.23	1.41	0.42	1.63	0.47	0.36	0.52	0.75	0.05	3.47	1.85	0.22	3.09	3847	109	19876400	4.10	56.38	39.52	41904178
Taizhoujs	江苏泰州	25.4	504.65	37.15	0.24		15.30	0.62	2.00	1.01	0.51	1.71	0.36	1.71	0.48	0.86	0.36	0.58	0.06	5.02	2.33	0.18	3.82	5787	65	20487200	7.40	54.95	37.64	49160775
Suqian	江苏宿迁	6.5	546.28	21.51	0.11	0.16	5.93	0.37	2.36	0.32	0.25	0.50	0.03	0.62	0.09	0.02	0.10	0.57	0.01	5.85	1.02	0.12	3.08	8555	65	10640900	17.58	45.03	37.39	11373660
Hangzhou	浙江杭州	84.6	689.12	232.71	0.15	0.27	75.91	2.19	47.49	8.78	6.49	12.85	8.10	7.52	6.05	7.42	8.52	4.38	0.83	14.41	7.76	2.03	11.56	16596	413	59491687	3.50	47.81	48.69	110796496
Ningbo	浙江宁波	102.1	574.08	140.19	0.13	0.02	63.67	1.76	26.62	4.65	0.92	5.03	1.79	5.25	1.87	5.24	1.33	1.29	0.31	7.42	4.58	1.01	7.30	9816	272	51630017	4.24	55.60	40.15	106187425
Wenzhou	浙江温州	29.5	786.80	107.92	0.11	0.22	50.70	1.38	20.21	2.97	0.54	2.33	1.26	2.90	1.53	1.33	0.67	0.44	0.08	8.71	4.03	0.80	7.71	11786	175	29250426	3.20	52.43	44.37	44948701
Jiaxing	浙江嘉兴	37.4	341.60	80.40	0.09		52.20	1.53	2.75	1.17	0.45	2.50	0.94	1.94	1.34	2.12	0.73	0.73	0.12	4.84	2.51	0.41	4.04	3915	85	23002027	5.52	58.24	36.24	51028508
Huzhou	浙江湖州	24.4	259.98	37.91	0.02	0.64	19.17	0.59	4.66	0.72	0.29	0.98	0.38	1.30	0.40	0.32	0.40	0.53	0.02	2.81	1.52	0.19	2.94	5818	78	13017294	8.01	54.93	37.07	26665326
Shaoxing	浙江绍兴	39.1	438.91	107.13	0.03	0.22	37.50	1.16	46.14	1.44	0.49	2.20	0.76	1.95	0.51	1.20	0.56	0.76	0.04	5.20	2.71	0.35	3.89	8279	100	27952029	5.35	56.05	38.60	67971868
Jinhua	浙江金华	43.1	466.65	52.50	0.10	0.05	15.39	0.92	10.11	1.72	0.62	1.90	0.82	2.23	0.47	1.41	0.46	1.24	0.03	5.43	2.87	0.44	6.29	10941	72	21100441	5.12	51.47	43.41	34117890
Zhoushan	浙江舟山	6.1	96.77	16.80	0.05	0.15	4.40	0.51	1.57	1.23	0.23	0.44	0.41	0.68	0.50	1.29	0.25	0.28	0.03	1.38	0.91	0.18	2.31	1440	52	6443170	9.63	45.52	44.85	9790517
Taizhouzj	浙江台州	30.8	583.14	69.78	0.48	0.02	22.87	1.05	18.86	1.34	0.60	2.00	0.80	3.40	0.76	1.39	0.65	0.71	0.14	5.92	3.07	0.37	5.35	9411	116	24264533	6.61	51.69	41.69	36307993
Lishui	浙江丽水	9.0	259.65	16.81	0.24	0.09	3.37	0.80	0.92	0.65	0.31	0.38	0.17	0.99	0.11	0.41	0.26	0.38	0.02	2.74	1.42	0.25	3.30	17298	32	6632932	9.49	49.54	40.97	11390737
Hefei	安徽合肥	34.1	494.95	71.10	0.10		17.79	0.89	14.25	4.52	0.97	4.62	1.28	2.40	1.59	1.03	2.60	1.05	0.18	7.66	3.25	1.04	5.88	7047	326	27016100	4.91	53.92	41.17	37990180
Wuhu	安徽芜湖	28.6	229.50	26.71	0.04	0.04	11.34	0.49	3.69	1.78	0.19	0.56	0.30	0.85	0.19	0.09	0.51	0.42	0.02	2.53	1.27	0.09	2.31	3317	135	11085924	4.44	65.18	30.38	22510125
Bengbu	安徽蚌埠	12.4	362.23	17.25	0.21		4.46	0.57	0.86	1.13	0.15	0.57	0.11	0.90	0.16	0.19	0.60	0.47	0.03	3.17	1.30	0.15	2.22	5941	105	6368877	18.84	47.25	33.91	7726842
Huainan	安徽淮南	53.3	243.99	32.70	0.32	12.99	3.46	1.55	2.90	0.96	0.13	0.64	0.24	0.84	0.89	1.24	0.34	0.54	0.02	2.63	1.16	0.13	1.72	2585	97	6035491	7.78	64.42	27.80	7889304
Maanshan	安徽马鞍山	55.4	129.10	15.42	0.03	1.48	6.93	0.38	0.89	0.34	0.07	0.36	0.04	0.61	0.04	0.32	0.26	0.17	0.01	1.38	0.62	0.12	1.37	1686	78	8110148	3.51	69.49	27.00	13575819
Huaibei	安徽淮北	38.6	219.56	20.84		11.31	2.82	0.50	0.27	0.41	0.10	0.16	0.06	0.36	0.03	0.07	0.11	0.12	0.02	2.03	0.81	0.06	1.60	2741	63	4616043	8.76	64.64	26.61	8814120
Tongling	安徽铜陵	18.9	74.01	11.67	0.38	0.12	5.00	0.29	2.05	0.20	0.08	0.24	0.07	0.32	0.10	0.07	0.13	0.13		0.82	0.38	0.08	1.21	1113	48	4667000	2.07	72.74	25.19	11042303
Anqing	安徽安庆	18.2	615.62	24.24	1.69	0.07	3.09	1.26	1.21	0.77	0.37	0.94	0.19	1.05	0.24	0.18	0.52	0.38	0.04	5.82	1.86	0.34	4.22	15318	77	9881100	15.74	53.03	31.23	13159401
Huangshan	安徽黄山	1.6	148.05	9.41	0.10	0.01	1.11	0.20	1.11	0.41	0.20	0.20	0.51	0.64	0.12	0.08	0.16	0.25	0.02	1.43	0.72	0.10	2.04	9807	44	3093198	12.72	44.09	43.20	3306897
Chuzhou	安徽滁州	11.4	450.80	17.85	0.85	0.34	3.18	0.32	1.26	0.97	0.19	0.52	0.10	0.76	0.09	0.09	0.19	0.46	0.02	3.86	1.34	0.09	3.22	13523	60	6956502	21.34	49.16	29.50	10529700
Fuyang	安徽阜阳	13.0	1011.84	29.11	0.31	1.60	3.66	0.74	3.03	1.33	0.27	1.46	0.15	1.61	0.27	0.26	0.18	0.48	0.02	6.83	2.11	0.17	4.63	9775	76	7218144	27.34	39.17	33.49	7037012
Suzhouah	安徽宿州	20.7	642.07	22.05	0.58	3.13	2.25	0.52	1.91	0.62	0.24	0.82	0.07	0.94	0.17	0.07	0.33	0.34	0.04	5.35	1.43	0.17	3.07	9787	53	6505700	27.89	37.88	34.23	6769181
Chaohu	安徽巢湖	20.1	460.51	16.89	0.21	0.17	2.16	0.40	2.75	0.30	0.16	0.75	0.20	0.56	0.20	0.35	0.20	0.30	0.02	3.82	1.28	0.10	2.96	9394	39	6297332	18.64	49.46	31.90	8261743
Luan	安徽六安	8.4	704.82	22.17	0.84	0.03	2.14	0.68	4.06	0.69	0.22	0.69	0.07	0.75	0.17	0.07	0.21	0.66	0.04	5.28	1.66	0.21	3.70	17976	61	6761209	23.56	42.26	34.18	8327000
Bozhou	安徽亳州	6.9	600.76	16.07	0.05	0.69	2.38	0.25	1.22	0.29	0.23	1.09	0.09	0.94	0.17	0.06	0.11	0.26	0.04	4.47	1.16	0.16	2.41	8374	57	5127800	26.75	37.36	35.90	3243341
Xuancheng	安徽宣城	11.7	278.36	12.34	0.29	0.01	3.20	0.38	0.28	0.30	0.15	0.19	0.06	0.57	0.32	0.27	0.14	0.12	0.01	2.21	1.07	0.11	2.66	12323	43	5257000	16.83	47.21	35.95	10677643
Fuzhoufj	福建福州	38.0	645.90	105.47	0.73	0.14	39.80	1.62	14.34	3.92	1.19	3.91	2.10	3.06	2.67	6.11	3.06	1.34	0.28	9.51	3.88	1.47	6.34	13066	220	31234092	9.05	44.88	46.06	45454115
Xiamen	福建厦门	11.8	180.21	95.33	0.28	0.01	52.41	0.78	12.82	4.04	1.13	3.32	2.60	1.39	3.68	2.00	0.54	0.66	0.82	3.71	1.81	0.57	2.76	1573	230	20600737	1.12	49.73	49.15	36889483
Putian	福建莆田	6.9	323.54	28.78	0.13	0.12	15.72	0.56	2.04	0.45	0.22	0.55	0.30	0.86	0.27	0.58	0.15	0.17	0.03	3.78	0.98	0.20	1.67	4119	55	8503257	10.33	56.11	33.56	12665314
Quanzhou	福建泉州	39.5	685.27	142.17	0.38	1.19	92.56	1.36	21.26	1.71	0.60	2.24	1.30	1.87	0.92	0.55	0.23	0.26	0.09	8.62	2.04	0.32	4.67	11015	160	35649739	3.71	60.16	36.13	62604054
Nanping	福建南平	7.5	313.90	23.54	1.09	0.21	7.68	0.89	0.83	0.68	0.37	0.74	0.37	1.13	0.37	0.35	0.35	0.54	0.05	3.45	1.48	0.28	2.68	26308	26	7286525	21.89	41.83	36.28	7774451
Longyan	福建龙岩	33.6	314.37	30.55	0.52	2.13	7.72	0.86	5.12	0.65	0.30	0.82	0.25	1.30	0.26	1.75	0.34	0.24	0.03	3.70	1.47	0.20	2.89	19063	38	9908973	13.01	53.25	33.74	11773399
Ningde	福建宁德	10.3	339.37	16.55	0.33	0.06	1.84	1.11	1.34	0.72	0.28	0.63	0.21	1.01	0.14	0.18	0.24	0.34	0.05	3.72	1.33	0.16	2.86	13452	19	7386099	18.50	42.95	38.56	9174539
Nanchang	江西南昌	39.9	502.25	67.83	1.73		17.16	1.62	13.33	7.86	0.67	1.49	0.32	2.23	0.43	0.74	1.48	1.61	0.16	7.23	3.03	1.25	5.49	7402	208	22001059	5.48	53.27	41.25	27732021
Jingdezhen	江西景德镇	12.1	163.16	17.44	1.02	0.61	6.53	0.44	1.22	0.41	0.12	1.08	0.10	0.50	0.20	0.15	0.33	0.13	0.03	1.79	0.66	0.18	1.94	5256	73	4615001	8.25	60.78	30.97	6871690
Pingxiang	江西萍乡	26.0	188.09	14.11	0.07	2.52	3.33	0.43	0.57	0.27	0.18	0.14	0.06	0.58	0.10	0.04	0.14	0.35	0.01	2.00	0.79	0.08	2.45	3824	42	5203900	8.13	63.31	28.56	11979370
Jiujiang	江西九江	24.9	497.91	33.95	0.89	0.24	8.91	1.26	5.03	0.84	0.35	0.61	0.25	1.12	0.24	0.99	0.66	0.46	0.08	4.91	1.92	0.31	4.88	18823	89	10320647	9.50	56.17	34.33	14977463
Xinyu	江西新余	29.4	118.01	10.07	0.09	0.38	4.48	0.47	0.46	0.21	0.11	0.13	0.04	0.33	0.02	0.02	0.10	0.21	0.03	1.24	0.43	0.06	1.26	3178	53	6312212	6.00	63.90	30.10	11987770
Yingtan	江西鹰潭	4.8	121.92	10.09	1.43	0.02	3.21	0.37	0.76	0.13	0.04	0.18	0.15	0.29	0.06	0.06	0.28	0.23		1.12	0.37	0.13	1.26	3560	29	3448865	9.51	62.78	27.71	11802016
Ganzhou	江西赣州	15.2	907.27	42.43	0.76	1.33	12.16	1.39	1.86	0.90	0.35	0.63	0.23	1.59	0.33	0.27	0.51	0.87	0.03	9.01	2.77	0.31	7.13	39379	76	11197412	18.92	44.36	36.72	12746325
Jian	江西吉安	9.6	495.04	20.15	1.39	0.57	1.19	1.02	1.09	1.01	0.34	0.61	0.16	0.92	0.21	0.20	0.32	0.51	0.01	4.51	1.66	0.22	4.21	25283	35	7205251	19.85	50.48	29.67	11349323
Yichunjx	江西宜春	24.6	557.93	28.07	0.78	2.67	6.11	0.94	1.09	0.98	0.36	0.76	0.20	1.08	0.21	0.19	0.22	0.48	0.02	4.84	1.96	0.19	4.99	18669	50	8700005	18.96	56.58	24.47	12399826
Fuzhoujx	江西抚州	4.1	403.96	21.17	0.98	0.10	4.18	0.72	2.38	0.50	0.29	0.79	0.04	0.71	0.09	0.07	0.18	0.38	0.01	4.26	1.15	0.17	4.17	18820	50	6300124	19.02	49.91	31.06	7347415
Shangrao	江西上饶	17.2	740.33	29.96	2.95	0.55	4.32	0.83	1.62	0.62	0.50	1.45	0.14	1.16	0.23	0.21	0.15	0.36	0.13	6.79	2.02	0.25	5.68	22791	38	9010029	16.86	50.96	32.18	11759109
Jinan	山东济南	64.9	604.08	127.80	0.11	1.85	30.76	1.89	27.43	8.88	1.73	7.03	2.60	5.94	2.83	2.84	2.50	1.32	1.14	10.35	4.64	1.64	12.32	8177	347	39105271	5.50	41.87	52.62	44856080
Qingdao	山东青岛	84.7	763.64	124.11	0.52	0.20	66.60	2.12	6.69	6.31	0.62	3.91	2.06	3.92	1.94	1.56	1.78	1.52	0.16	11.05	4.39	1.06	7.70	10978	282	56661900	4.89	48.69	46.43	106628345
Zibo	山东淄博	89.2	422.36	62.88	0.25	4.46	26.93	1.56	7.58	0.95	0.37	2.90	0.69	1.27	0.68	0.56	0.24	0.58	0.04	5.74	2.49	0.50	5.09	5965	225	28667500	3.67	61.62	34.70	77423440
Dongying	山东东营	51.1	184.87	40.10	0.59	12.25	9.02	0.28	1.35	1.38	0.51	0.70	0.66	0.72	0.16	1.31	1.72	0.33	1.46	3.11	1.07	0.11	3.37	7923	108	23599400	3.70	72.55	23.74	60378601
Weifang	山东潍坊	67.9	873.78	73.99	0.34	1.16	30.95	1.61	4.65	1.20	0.49	4.30	0.69	1.84	0.66	0.38	0.69	0.82	0.07	10.41	4.51	0.33	8.89	16140	140	30909200	10.69	55.66	33.65	75292226
Taian	山东泰安	63.9	557.01	56.32	0.26	11.19	14.29	1.05	7.82	1.28	0.42	2.52	0.57	1.44	0.55	0.34	0.59	0.57	0.14	5.92	2.39	0.29	4.69	7762	107	20516800	9.52	53.59	36.89	37700720
Linyi	山东临沂	59.5	1072.69	56.12	0.70	2.40	16.60	1.30	4.30	0.90	0.40	2.00	0.30	2.60	0.20	0.40	0.40	0.70	0.02	10.10	3.70	0.30	8.80	17191	166	23999900	11.00	50.26	38.74	45935251
Heze	山东菏泽	20.7	958.80	37.21	0.42	0.66	6.19	1.25	1.82	1.10	0.15	1.30	0.24	1.35	0.10	0.34	0.22	0.94	0.03	8.60	3.29	0.27	8.94	12239	77	12270900	17.94	52.85	29.20	25320325
Zhengzhou	河南郑州	76.3	963.00	108.50	0.23	7.17	20.54	3.11	19.57	3.00	1.00	5.14	2.89	4.15	2.42	2.13	2.96	1.75	0.31	12.81	5.28	2.08	11.96	7446	343	40408926	3.08	56.17	40.74	59137624
Luoyang	河南洛阳	67.0	703.54	53.92	0.21	1.51	14.83	3.66	3.81	1.81	0.20	2.29	0.71	1.99	0.48	0.94	2.25	1.02	0.21	7.31	3.11	0.47	7.11	15200	181	23202460	8.09	60.18	31.74	41323063
Pingdingshan	河南平顶山	59.7	539.59	48.45	0.12	11.83	11.05	1.08	2.82	1.12	0.15	2.10	0.47	1.66	0.30	0.96	0.40	0.73	0.03	5.40	2.04	0.34	5.85	7904	71	13108394	8.75	66.33	24.92	20031593
Hebi	河南鹤壁	23.9	162.05	17.81	0.12	4.78	4.41	0.34	2.15	0.20	0.08	0.48	0.14	0.32	0.19	0.05	0.09	0.31		1.68	0.59	0.09	1.79	2182	51	4291193	11.38	70.37	18.26	9890970
Xinxiang	河南新乡	32.4	603.86	45.69	0.63	0.40	14.67	0.97	5.44	0.93	0.15	2.17	0.41	1.03	0.45	0.45	0.71	0.53	0.10	6.82	2.76	0.30	6.77	8169	97	11899408	13.21	57.69	29.10	21733234
Jiaozuo	河南焦作	37.7	368.02	32.29	0.57	3.03	9.94	1.28	1.74	0.59	0.17	1.18	0.41	1.70	0.11	0.20	0.26	0.65	0.04	3.94	1.70	0.21	4.57	4071	90	12459260	8.13	68.65	23.22	28858779
Puyang	河南濮阳	16.9	409.83	31.42	0.05	6.68	3.04	0.67	6.10	0.91	0.10	0.86	0.20	0.78	0.17	1.48	0.15	0.43	0.12	4.29	1.33	0.20	3.86	4266	51	7754037	13.88	66.46	19.66	13852696
Xuchang	河南许昌	29.8	489.64	28.67	0.06	2.15	8.07	0.67	2.43	0.65	0.10	0.73	0.25	0.54	0.31	0.44	0.28	0.44	0.03	4.61	1.87	0.27	4.77	4996	80	13164870	11.39	68.51	20.10	24966545
Sanmenxia	河南三门峡	112.7	230.30	24.22	0.14	5.97	3.77	0.80	2.32	0.63	0.13	1.68	0.28	0.80	0.08	0.35	0.22	0.19	0.03	2.80	0.97	0.16	2.90	10496	30	8744157	8.01	68.52	23.47	20398477
Nanyang	河南南阳	155.7	1186.69	70.48	1.16	3.26	16.37	1.60	6.80	2.18	0.63	5.59	0.91	1.94	0.75	1.40	1.28	1.31	0.18	12.16	3.94	0.56	8.46	26509	92	19533562	20.54	52.07	27.39	24952130
Shangqiu	河南商丘	46.9	918.01	39.72	0.28	3.55	3.23	0.77	4.06	1.05	0.27	1.93	0.28	1.02	0.13	0.13	0.25	0.60	0.04	9.52	3.10	0.20	9.31	10704	60	11437913	26.19	46.52	27.29	13594361
Xinyang	河南信阳	19.8	870.22	43.19	0.88	0.54	5.98	1.40	5.59	1.58	0.40	3.12	0.54	1.23	0.58	0.52	0.92	0.93	0.06	9.28	2.48	0.41	6.75	18847	68	10918323	26.38	42.21	31.41	11175624
Zhoukou	河南周口	44.6	1224.35	45.22	1.10		6.31	1.25	4.87	0.96	0.45	3.13	0.27	1.79	0.51	0.16	0.19	0.53	0.03	10.70	2.98	0.29	9.70	11959	51	12283024	29.77	45.42	24.81	15007129
Zhumadian	河南驻马店	71.5	886.10	41.16	0.52	0.19	6.88	1.28	6.06	1.14	0.31	2.70	0.35	0.97	0.97	0.34	0.56	0.63	0.16	8.17	2.94	0.34	6.65	15083	52	10537118	27.58	41.88	30.54	11377676
Wuhan	湖北武汉	101.4	836.73	178.46	0.79	0.08	50.26	2.00	36.70	14.92	2.22	12.30	4.69	5.50	2.92	2.11	5.74	2.31	0.71	16.90	6.51	2.06	9.74	8494	500	55659300	3.06	45.51	51.44	64245900
Huangshi	湖北黄石	30.0	260.14	43.85	0.44	5.65	16.67	0.65	6.73	0.98	0.19	1.55	1.05	0.52	0.83	0.21	0.50	0.51	0.40	2.86	1.08	0.52	2.51	4586	66	6901200	7.77	57.22	35.01	11975700
Shiyan	湖北十堰	20.1	353.19	43.26	0.36	0.47	17.38	1.41	2.24	1.02	0.85	5.65	0.70	0.75	0.68	0.57	0.39	0.29	0.42	3.84	1.87	0.64	3.73	23680	62	7367800	10.56	54.57	34.87	12804019
Yichang	湖北宜昌	25.0	398.55	55.73	0.28	2.31	18.74	2.95	7.27	3.03	0.53	4.46	1.17	0.95	1.26	1.12	0.80	0.52	0.31	3.79	2.27	0.51	3.46	21084	92	15473200	11.41	57.53	31.07	21187359
Xiangyang	湖北襄阳	22.0	591.07	46.53	0.60	0.35	17.36	0.99	4.07	1.31	0.27	1.65	0.47	1.41	0.38	0.16	1.11	1.25	0.06	6.12	2.77	0.32	5.88	19724	107	15382700	15.26	51.89	32.85	22988086
Ezhou	湖北鄂州	24.2	108.46	18.22	0.03	0.80	7.50	0.26	3.46	0.43	0.10	0.98	0.38	0.34	0.32	0.24	0.14	0.26	0.07	1.32	0.52	0.11	0.96	1594	52	3952900	13.02	58.53	28.46	6330996
Jinmen	湖北荆门	26.1	300.40	31.44	1.08	1.12	11.83	0.65	2.34	0.84	0.46	2.22	0.42	0.78	0.61	0.70	0.36	0.44	0.47	3.10	1.38	0.20	2.44	12404	50	7300700	19.87	48.37	31.76	12001085
Jinzhou	湖北荆州	11.9	658.17	42.72	3.97	0.16	14.82	0.80	3.07	1.01	0.28	0.85	0.35	1.11	0.33	0.26	0.52	0.70	0.06	5.18	3.18	0.32	5.75	14092	66	8371000	27.60	38.86	33.53	8975700
Xianning	湖北咸宁	11.1	290.96	19.58	0.39	0.15	5.64	0.48	0.98	0.54	0.12	0.33	0.27	0.52	0.25	0.26	0.45	0.64	0.08	3.36	1.58	0.11	3.43	9861	63	5203300	19.41	45.70	34.90	6333953
Suizhou	湖北随州	3.2	257.91	10.92	0.06	0.11	2.82	0.13	1.57	0.25	0.07	0.40	0.14	0.30	0.06	0.02	0.10	0.37	0.01	1.96	0.89	0.09	1.57	9636	43	4016600	21.55	45.23	33.22	5097629
Enshizhou	湖北恩施州	8.2																												
Changsha	湖南长沙	53.0	652.40	110.56	0.05	0.04	30.51	1.61	16.99	2.84	1.69	6.65	4.40	5.31	4.16	2.13	3.81	1.72	0.52	10.98	5.72	1.92	8.55	11816	272	45470573	4.44	53.60	41.96	41654294
Xiangtan	湖南湘潭	32.1	288.98	29.57	0.81		8.32	0.49	7.45	0.60	0.12	1.76	0.32	0.85	0.50	0.21	0.21	0.25	0.03	3.02	1.28	0.11	3.24	5015	73	8940050	10.74	55.86	33.40	14962525
Hengyang	湖南衡阳	15.4	791.62	51.69	0.03	0.12	9.69	1.01	10.95	1.30	0.41	1.06	0.73	1.90	0.74	0.58	0.70	0.75	0.07	7.71	3.26	0.42	8.07	15299	99	14203377	18.62	45.46	35.92	18832469
Shaoyang	湖南邵阳	14.6	793.97	33.47	0.56	1.37	2.96	0.96	6.75	1.48	0.38	0.59	0.12	1.39	0.43	0.39	0.31	0.51	0.02	6.09	2.54	0.16	6.46	20830	49	7272893	23.89	38.23	37.89	7127007
Yueyang	湖南岳阳	21.4	565.62	55.02	3.23	0.01	18.30	0.75	7.76	1.67	0.46	1.71	0.90	1.53	0.68	0.66	0.43	0.64	0.35	5.48	2.26	0.28	7.27	15087	82	15393576	14.00	54.19	31.80	27843253
Changde	湖南常德	23.5	623.11	36.81	0.10	0.53	8.09	0.81	7.55	0.59	0.47	0.91	0.31	1.10	0.59	1.21	0.27	0.76	0.12	5.23	2.36	0.31	5.50	18190	76	14915686	18.78	45.94	35.28	12626665
Zhangjiajie	湖南张家界	4.6	164.75	7.93	0.07	0.18	0.35	0.34	0.83	0.34	0.18	0.11	0.31	0.34	0.06	0.07	0.12	0.37	0.01	1.59	0.69	0.06	1.91	9516	28	2424785	12.88	24.77	62.35	1089444
Yiyang	湖南益阳	15.3	476.36	24.07	0.15		4.30	0.40	3.19	0.35	0.24	0.40	0.26	1.30	0.41	0.67	0.30	0.47	0.08	3.90	1.84	0.14	5.53	12144	54	7122748	22.80	40.49	36.71	8080903
Chenzhou	湖南郴州	28.8	502.07	28.95	0.10	3.55	4.16	1.33	1.96	0.71	0.31	0.90	0.50	0.66	0.41	0.41	0.31	0.54	0.04	4.57	2.20	0.28	6.01	19699	62	10817632	11.72	54.95	33.33	14400825
Huaihua	湖南怀化	15.3	509.72	25.62	0.40		2.61	1.39	1.58	1.27	0.40	0.37	0.28	1.04	0.30	0.45	0.40	0.70	0.11	5.06	2.27	0.26	6.35	27624	52	6749227	14.44	42.80	42.76	6998921
Xiangxi	湖南湘西	3.6																												
Guangzhou	广东广州	100.5	806.14	246.37	0.60	0.04	88.64	2.44	14.61	22.57	5.29	12.21	10.11	7.96	9.13	9.93	7.68	3.36	3.05	18.31	11.20	3.61	15.63	7434	952	107482828	1.75	37.24	61.01	138312477
Shaoguan	广东韶关	22.7	328.10	30.61	0.46	0.89	10.24	1.38	2.82	1.30	0.26	0.52	0.38	0.88	0.33	0.71	0.34	0.63	0.04	3.86	1.57	0.15	3.85	18463	78	6831033	14.04	41.78	44.18	7733678
Shenzhen	广东深圳	38.6	259.87	253.02	0.27	0.18	123.66	1.87	13.00	16.31	5.19	13.76	7.08	10.86	12.69	12.42	5.43	1.81	1.94	7.66	5.38	1.70	11.81	1992	830	95815101	0.07	47.21	52.72	185268200
Zhuhai	广东珠海	13.0	104.74	63.15	0.74	0.03	41.76	0.45	1.93	1.45	0.88	2.13	1.27	1.85	1.39	0.93	0.42	0.81	0.18	2.25	1.09	0.45	3.14	1711	124	12085958	2.68	54.77	42.55	29761820
Shantou	广东汕头	24.4	524.11	32.28	0.04	0.02	9.47	0.65	3.13	1.04	0.45	1.99	0.56	1.42	0.37	0.34	0.37	0.63	0.10	5.85	1.98	0.23	3.64	2064	182	12089743	5.34	56.10	38.56	18975666
Jiangmen	广东江门	30.9	392.28	44.85	0.11		22.65	0.80	3.54	0.91	0.43	1.06	0.75	1.80	0.39	0.30	0.24	0.59	0.19	4.47	2.27	0.20	4.15	9568	129	15704191	7.45	55.54	37.01	38289057
Maoming	广东茂名	27.8	747.17	30.94	1.19	0.30	3.69	0.72	4.75	0.90	0.29	0.80	0.26	0.98	0.27	0.52	0.17	0.63	0.24	8.69	2.14	0.41	3.99	11458	70	14920857	18.40	39.59	42.01	13601493
Huizhou	广东惠州	23.6	337.28	80.39	0.11	0.03	57.55	0.73	2.19	1.25	0.35	1.10	0.79	1.87	0.89	0.73	0.42	0.72	0.14	4.09	1.86	0.36	5.21	11343	215	17299543	5.92	58.94	35.15	39051731
Heyuan	广东河源	9.4	358.39	24.68	0.13	0.28	11.02	0.71	1.09	0.64	0.16	0.46	0.30	0.58	0.24	0.42	0.21	0.24	0.05	3.69	1.19	0.15	3.09	15642	29	4751396	12.72	51.45	35.83	8327317
Yangjiang	广东阳江	15.8	282.81	18.24	0.57	0.03	2.79	0.47	3.20	0.59	0.29	0.96	0.26	0.58	0.56	0.26	0.15	0.37	0.05	3.04	1.17	0.12	2.78	7946	44	6398389	21.92	42.45	35.62	6934570
Zhongshan	广东中山	17.2	149.18	29.04			17.29	0.42	0.28	1.03	0.28	0.34	0.33	1.34	0.42	0.50	0.22	0.13	0.04	2.46	1.48	0.21	2.27	1800	41	18506521	2.74	58.04	39.22	50236309
Yunfu	广东云浮	17.9	282.76	17.48	0.07	0.33	6.81	0.46	0.60	0.24	0.20	0.90	0.14	0.48	0.11	0.10	0.09	0.20	0.04	3.02	0.91	0.11	2.67	7779	19	4009741	25.12	41.18	33.70	4663419
Nanning	广西南宁	23.4	707.37	70.55	1.54	0.15	14.43	1.11	6.78	3.18	0.90	4.50	1.43	2.62	1.64	3.23	2.54	1.51	0.16	9.77	4.43	1.14	9.49	22112	215	18002613	13.58	36.21	50.21	12854044
Liuzhou	广西柳州	48.9	372.69	37.99	0.79	0.25	13.26	0.67	2.31	1.59	0.49	1.49	0.52	1.21	0.54	2.25	0.66	1.16	0.24	4.48	2.37	0.28	3.43	18617	135	13153121	8.32	63.86	27.82	23887937
Guigang	广西贵港	36.8	523.81	15.31	0.21	0.04	2.49	0.40	0.66	0.75	0.07	0.49	0.01	0.45	0.04	0.15	0.25	0.42	0.02	5.01	1.42	0.05	2.38	10602	56	5446571	19.84	45.58	34.58	4702986
Laibin	广西来宾	21.1	260.10	11.85	0.91	0.56	2.24	0.58	0.32	0.21	0.14	0.28	0.03	0.31	0.07	0.20	0.32	0.31	0.01	2.57	1.02	0.08	1.69	13411	29	4048883	24.16	47.42	28.41	3682803
Chongqing	重庆	147.5	3303.45	248.75	1.71	9.53	57.42	6.42	43.32	13.45	2.55	11.17	4.25	9.75	4.88	4.30	5.39	3.38	0.77	33.78	10.89	2.41	23.38	82829	870	79255800	8.65	55.00	36.35	91435532
Chengdu	四川成都	41.6	1149.07	172.05	0.23	0.07	45.19	2.16	43.49	5.42	1.31	8.73	3.12	5.23	2.30	2.97	6.85	2.39	0.50	16.32	8.70	1.83	15.24	12132	456	55513336	5.14	44.69	50.17	58097349
Zigong	四川自贡	11.5	325.96	16.54	0.06	0.62	3.65	0.40	2.54	0.84	0.24	0.39	0.10	0.98	0.30	0.07	0.24	0.25	0.03	2.42	1.28	0.11	2.02	4373	80	6477251	13.07	57.25	29.67	11081674
Panzhihua	四川攀枝花	81.2	111.38	17.18	0.12	1.90	7.99	0.47	1.12	0.36	0.12	0.21	0.08	0.58	0.04	0.07	0.11	0.26	0.01	1.31	0.62	0.12	1.69	7440	55	5239883	4.10	73.79	22.11	9532489
Deyang	四川德阳	12.2	389.15	25.74	0.07	0.49	8.01	0.34	5.26	0.57	0.15	0.40	0.11	1.21	0.08	0.10	0.26	0.54	0.03	3.38	1.58	0.09	3.07	5911	54	9212679	16.54	57.82	25.63	16014090
Mianyang	四川绵阳	16.6	541.87	34.56	0.16	0.01	12.11	0.87	3.32	0.94	0.29	0.65	0.22	1.54	0.27	0.15	1.99	0.77	0.04	5.03	1.94	0.16	4.10	20249	103	9602153	17.34	48.77	33.89	12675301
Guiyang	贵州贵阳	40.4	337.16	70.34	0.30	1.01	14.90	1.42	16.79	2.44	1.14	5.13	1.74	1.77	2.60	1.86	1.68	1.04	0.73	5.92	2.80	0.93	6.14	8034	162	11218174	5.09	40.73	54.18	10583479
Zunyi	贵州遵义	23.6	784.16	30.34	0.13	0.26	5.51	1.00	1.53	1.42	0.30	1.30	0.11	1.00	0.23	0.38	0.59	0.68	0.10	7.16	2.00	0.18	6.46	30762	47	9087570	15.43	41.78	42.79	7937190
Kunming	云南昆明	80.9	583.99	98.96	0.80	1.49	21.21	1.54	14.62	8.24	1.76	7.05	2.85	2.96	2.10	3.59	3.56	1.21	0.59	9.29	4.20	1.56	9.54	21015	275	21203700	5.67	45.32	49.01	24361300
Xian	陕西西安	55.7	782.73	140.37	0.39	0.37	42.00	3.23	11.62	9.99	4.94	6.19	4.16	5.89	3.40	1.65	9.07	2.27	1.47	16.15	5.58	2.53	9.47	10108	327	32414900	4.32	43.48	52.20	31253569
Baoji	陕西宝鸡	26.6	381.09	31.65	0.50	0.65	10.85	0.92	2.08	1.98	0.34	1.71	0.32	0.89	0.14	0.11	0.50	0.51	0.05	4.42	1.99	0.22	3.47	18131	118	9760900	10.68	62.95	26.38	13168575
Xianyang	陕西咸阳	26.8	520.09	37.10	0.38	1.49	9.15	1.14	3.26	0.77	0.33	0.92	0.33	1.31	0.24	0.42	0.70	1.21	0.03	7.19	2.24	0.36	5.63	10196	81	10986810	18.50	52.18	29.32	13353500
Yulin	陕西榆林	54.1	364.50	25.91	0.62	3.78	1.40	1.94	0.56	1.16	0.19	0.65	0.09	0.65	0.13	0.24	0.38	1.23	0.01	4.69	1.52	0.30	6.37	43578	52	17566680	5.25	68.64	26.11	19599930
Lanzhou	甘肃兰州	43.1	323.54	51.36	0.16	1.35	12.50	1.53	8.74	1.75	0.54	1.90	0.70	2.10	0.59	1.17	2.59	0.89	0.08	5.68	1.97	1.05	6.07	13086	196	11003898	3.07	48.09	48.84	15926000
Jiayuguan	甘肃嘉峪关	22.9	21.80	5.01	0.01	0.01	3.55	0.10	0.02	0.04	0.04	0.05	0.04	0.13	0.01	0.04	0.02	0.12	0.01	0.23	0.12	0.05	0.42	2935	50	1843192	1.34	80.16	18.50	5155800
Baiyin	甘肃白银	22.0	180.39	15.34	0.46	2.49	3.81	0.59	0.40	0.37	0.12	0.32	0.03	0.51	0.03	0.05	0.15	0.21	0.07	2.74	0.37	0.07	2.55	21158	55	3111826	12.10	54.99	32.91	4299063
Wuwei	甘肃武威	4.9	191.26	9.89	1.12	0.34	1.24	0.38	0.38	0.46	0.06	0.22	0.04	0.48	0.26	0.27	0.22	0.35	0.01	1.68	0.74	0.28	1.36	33238	27	2287676	26.43	40.01	33.56	1755165
Xining	青海西宁	28.0	220.87	28.65	0.14	0.39	7.25	0.51	3.37	2.34	0.58	1.39	0.38	1.25	0.56	0.48	1.41	0.65	0.06	2.92	1.76	0.43	2.78	7655	67	6282800	3.89	51.05	45.05	7512207
Yinchuan	宁夏银川	95.5	158.80	30.08	1.22	5.15	4.68	2.48	1.64	0.90	0.41	0.85	0.24	1.68	0.49	1.02	0.94	0.70	0.02	2.65	1.37	0.50	3.14	9025	121	7694227	5.26	50.10	44.64	9355637
Urumchi	新疆乌鲁木齐	59.0	243.03	47.17	1.23	1.77	7.97	1.09	5.63	5.81	0.53	2.04	1.03	1.73	0.75	1.29	1.97	0.53	0.07	4.74	2.91	1.01	5.07	13788	343	13385172	1.49	44.86	53.65	16727154
Karamay	新疆克拉玛依	61.1	37.51	15.76	0.04	7.24	3.10	0.01	1.30	0.23	0.10	0.33	0.11	0.25	0.34	0.82	0.06	0.09	0.01	0.62	0.26	0.01	0.84	9548	57	7113531	0.49	89.75	9.76	13303969
Aletai	新疆阿勒泰	4.5	19.70	33677																				11481	10.6					83849.5
Bayinguoleng	新疆巴音郭楞	18.0																												
Tulufan	新疆吐鲁番	11.1	28	27135																				13589	13.4					372073
