# Supplementary material for: An emissions-socioeconomic inventory of Chinese cities
Source: Sci Data. 2019 Feb 26;6:190027. doi: 10.1038/sdata.2019.27 (PMC6390707; doi:10.1038/sdata.2019.27)
Supplement: Supplementary Information [file sdata201927-s2.docx]

**Supplementary Information of**

**An emissions-socioeconomic inventory of Chinese cities**

*Yuli Shan^1,2^, Jianghua Liu^3^, Zhu Liu^2,4^, Shuai Shao^3,*^, Dabo Guan^2,4,*^*

1. Water Security Research Centre and School of International Development, University of East Anglia, Norwich NR4 7TJ, UK
2. Tyndall Centre for Climate Change Research, University of East Anglia, Norwich NR4 7TJ, UK
3. School of Urban and Regional Science, School of Urban and Regional Science, Shanghai University of Finance and Economics, Shanghai 200433, China
4. Department of Earth System Science, Tsinghua University, Beijing 100080, China

Corresponding authors: Shuai Shao ([shao.shuai@shufe.edu.cn](mailto:shao.shuai@shufe.edu.cn)); Dabo Guan ([dabo.guan@uea.ac.uk](mailto:dabo.guan@uea.ac.uk))

**MATLAB code for cities’ emission inventory construction**

We take Beijing 2010 as an example to show the calculation of emission inventory with MATLAB R2014a using the energy data.

*% Read emission factors and energy data from the excel files*

NCV=xlsread('Emission factors', 'NCV', 'A2:Q2'); *%NCV_i_ refers to Table 1*

NCV=repmat(NCV,68,1);

CC=xlsread('Emission factors', 'CC', 'A2:Q2'); *%CC_i_ refers to Table 1*

CC=repmat(CC,47,1);

O = xlsread('Emission factors', 'Oxygenation Efficiency', 'B2:R48'); *%O_ij_ refers to Table 3*

Energy=xlsread(China city-level Energy inventory, 2010,'Beijing','B3:R70');

*% Convert physical energy consumption to calories*

E_PJ1 = Energy .* NCV;

E_PJ1(1,:) = sum(E_PJ1(2:48,:));

E_PJ2 = E_PJ1;

*% Remove non-energy use from the total consumption*

E_NE = zeros (68,17);

E_NE (23:27, 1:17) = repmat (E_PJ1(65,:),5,1) .* E_PJ2(23:27,:) ./ repmat (sum(E_PJ1(23:27,:)),5,1);

E_NE (:,16) = E_PJ2 (:,16);

E_NE(isnan(E_NE))=0;

*% Include energy combustion consumption during transformation process*

E_Trans = zeros (68,17);

E_Trans (40,:) = E_PJ1(52,:)+E_PJ1(53,:);

E_Trans (44,10:13) = E_PJ1(67,10:13)+E_PJ1(68,10:13);

E = E_PJ2(1:48,:)-E_NE(1:48,:)+E_Trans(1:48,:);

E(1,:) = sum(E(2:48,:));

*% Calculate the energy-related emissions*

CO2_E = E (2:48,:) .* CC .* O / 100;

CO2_E(CO2_E<0)=0;

*% Calculate the process-related emissions*

EF_cement=0.2906;

Prod_cement=54.0223; *%Prod_cement here refers to Beijing’s cement production in 2010*

CO2_cement = Prod_cement .* EF_cement;

*% Construct the emission inventory*

CO2 = zeros (48,19);

CO2 (2:48,1:17) = CO2_E;

CO2 (28,18)= CO2_cement;

CO2 (:, 19) = sum(CO2,2);

CO2 (1,:) = sum(CO2(2:48,:))
